# FBXO38 Ubiquitin Ligase Controls Sertoli Cell Maturation

**DOI:** 10.3389/fcell.2022.914053

**Published:** 2022-06-13

**Authors:** Nikol Dibus, Eliska Zobalova, Mario A. M. Monleon, Vladimir Korinek, Dominik Filipp, Jana Petrusova, Radislav Sedlacek, Petr Kasparek, Lukas Cermak

**Affiliations:** ^1^ Laboratory of Cancer Biology, Institute of Molecular Genetics of the Czech Academy of Sciences, Vestec, Czechia; ^2^ Faculty of Science, Charles University, Prague, Czechia; ^3^ Laboratory of Cell and Developmental Biology, Institute of Molecular Genetics of the Czech Academy of Sciences, Prague, Czechia; ^4^ Laboratory of Immunobiology, Institute of Molecular Genetics of the Czech Academy of Sciences, Prague, Czechia; ^5^ Czech Centre for Phenogenomics, Institute of Molecular Genetics of the Czech Academy of Sciences, Vestec, Czechia

**Keywords:** proteasome, ubiquitin, ubiquitin ligase, spermatogenesis, sertoli cell, centromere, retinoic acid

## Abstract

The ubiquitin ligase SCF^FBXO38^ controls centromeric chromatin by promoting the degradation of the ZXDB protein. To determine the importance of this pathway during development, *Fbxo38*-deficient mice were generated. The loss of FBXO38 resulted in growth retardation affecting several organs, including the male reproductive system. A detailed analysis of the mutant testes revealed pathological changes in the seminiferous tubules, accompanied by a significant decrease in sperm production and reduced fertility. In adult testes, FBXO38 was specifically expressed in Sertoli cells, a somatic population essential for spermatogenesis initiation and progression. Sertoli cells lacking FBXO38 exhibited stabilized ZXDB protein and upregulated centromeric chromatin. Furthermore, the gene expression profile revealed that the absence of FBXO38 led to a defect in Sertoli cell maturation, specifically characterized by dysregulation in genes controlling retinoic acid metabolism and intercellular communication. Consequently, we documented significant changes in their ability to initiate spermatogonial differentiation. In conclusion, we show that FBXO38 acts as a Sertoli cell maturation factor, affecting the Sertoli cell transcription program, centromere integrity, and, subsequently, the ability to control spermatogenesis.

## Introduction

F-box-containing protein 38 (FBXO38), a member of the F-box family, was initially discovered as a co-activator of the transcription factor Krüppel-like factor 7 (KLF7) (Smaldone et al., 2004). FBXO38 acts as a substrate-binding receptor for multi-subunit ubiquitin ligase SCF^FBXO38^ (SKP1-CUL1-FBXO38). In this complex, a CUL1 scaffold protein separates substrate recognition and ubiquitination modules. The protein adaptor SKP1 connects CUL1 to different F-box-containing proteins, thus providing versatility to the ubiquitin-mediated degradation system (Skaar et al., 2013; Baek et al., 2021). Although several studies showed FBXO38 might act both as a part or independently of the SCF complex, the only substrate of SCF^FBXO38^ identified, so far, was the T-cell-specific transmembrane receptor programmed cell death 1 (PD1) (Meng et al., 2018; Georges et al., 2019). Recently, we have shown that FBXO38 controls the stability of ZXDA/B zinc finger proteins. ZXDA/B are encoded by an intronless retroposed gene (retrogene) derived from their homolog ZXDC (Greig et al., 1993; Aleksandrova et al., 2010). In mouse genome, *Zxda* gene contains frameshift mutation and thus *Zxdb* gene is the only functional mouse ortholog. In addition, we discovered that FBXO38-dependent degradation of ZXDB was responsible for the dynamic control of the centromeric chromatin, particularly the centromeric protein CENP-B (manuscript co-submitted). Interestingly, mutations in the FBXO38 gene were found in three families with early-onset distal hereditary motor neuronopathy (Sumner et al., 2013; Akçimen et al., 2019). Other genetic studies showed FBXO38 gene polymorphism in patients suffering from severe chronic periodontitis and chronic obstructive pulmonary disease (Shang et al., 2015; Saferali et al., 2019). Moreover, a genetic analysis of monozygotic twins with discordant development of gender dysphoria revealed the potential role of FBXO38 in the control of gender identity (Morimoto et al., 2017).

Spermatogenesis represents a well-documented and straightforward system of stem cell differentiation. Spermatogonial stem cell division is controlled by a stem cell niche composed of cellular and cell-free components (Oatley and Brinster, 2012; Griswold, 2016). Spermatogonial precursor cells undergo several mitotic divisions, giving rise to spermatocytes that divide meiotically, resulting in the formation of haploid germ cells that are further transformed into mature spermatozoa. The process of spermatogenesis is localized to the seminiferous tubules arranged in the testes. Each tubule is surrounded by a basal lamina formed by an extracellular matrix with the peritubular myoid cells providing structural support. The interstitial space contains Leydig cells responsible for testosterone production. Inside the tubules, somatic Sertoli cells are essential for the whole process of spermatogenesis (Griswold, 2018). During embryogenesis, fetal Sertoli cells surround the gonocytes (spermatogonial precursors), proliferate, and drive the tubule formation. Interestingly, Sertoli cell proliferation occurs rarely after the onset of puberty when Sertoli cells undergo a complex change in gene expression and finally reach terminal differentiation (Figueiredo et al., 2016; Meroni et al., 2019). Since each Sertoli cell is capable of supporting a limited number of germ and Leydig cells, testis size is determined by the proliferation potential of the Sertoli cell population. In adults, Sertoli cells provide paracrine (niche) support to differentiating germ stem cells. Additionally, a “web” of interconnected Sertoli cells constitutes the blood-testis barrier (BTB). Moreover, the cells are involved in seminiferous tubule clearance from apoptotic and cytosolic residues (Wu et al., 2022). To control spermatogonial differentiation, Sertoli cells transmit signals generated by the hypothalamic-pituitary-Leydig cell system. Interestingly, the activity of this hormonal germ cell differentiation axis is negatively regulated by Sertoli cells through the secretion of serum inhibins (Krause, 1977). Upon activation, Sertoli cells produce several morphogens that provide paracrine control of spermatogenic events across the tubule. One of the most important morphogenic compounds is all-trans retinoic acid (ATRA), responsible for spermatogenesis initiation (Griswold, 2016). Serum retinol (or vitamin A) is actively transported to Sertoli cells, where it is metabolically modified into retinaldehyde and, finally, to ATRA. Upon release from the Sertoli cells, it enters spermatogonia and activates the pro-mitotic program. ATRA is actively degraded in Sertoli cells during early postnatal development of the testes by cytochrome P450 family 26 subfamily B member 1 (CYP26B1), the enzyme preventing premature initiation of differentiation. In the first wave of spermatogenesis, CYP26B1 activity ensures the coordinated entry of spermatogonia into meiosis, a process required for continuous sperm production in adulthood (MacLean et al., 2007; Griswold, 2016).

Here, we investigated the role of FBXO38 in mouse development. FBXO38 controls the growth of several organs, including testes, where it is specifically expressed by stem cell-supporting Sertoli cells. Sertoli cells lacking FBXO38 exhibit a defect in maturation resulting in improper spermatogonia stimulation and disrupted sperm production. This pathologic process is accompanied by stabilization of the FBXO38 substrate ZXDB and alteration in CENP-A/B positive centromeric regions.

## Results

### FBXO38 Deficiency Leads to Growth Retardation

**FIGURE 1:**
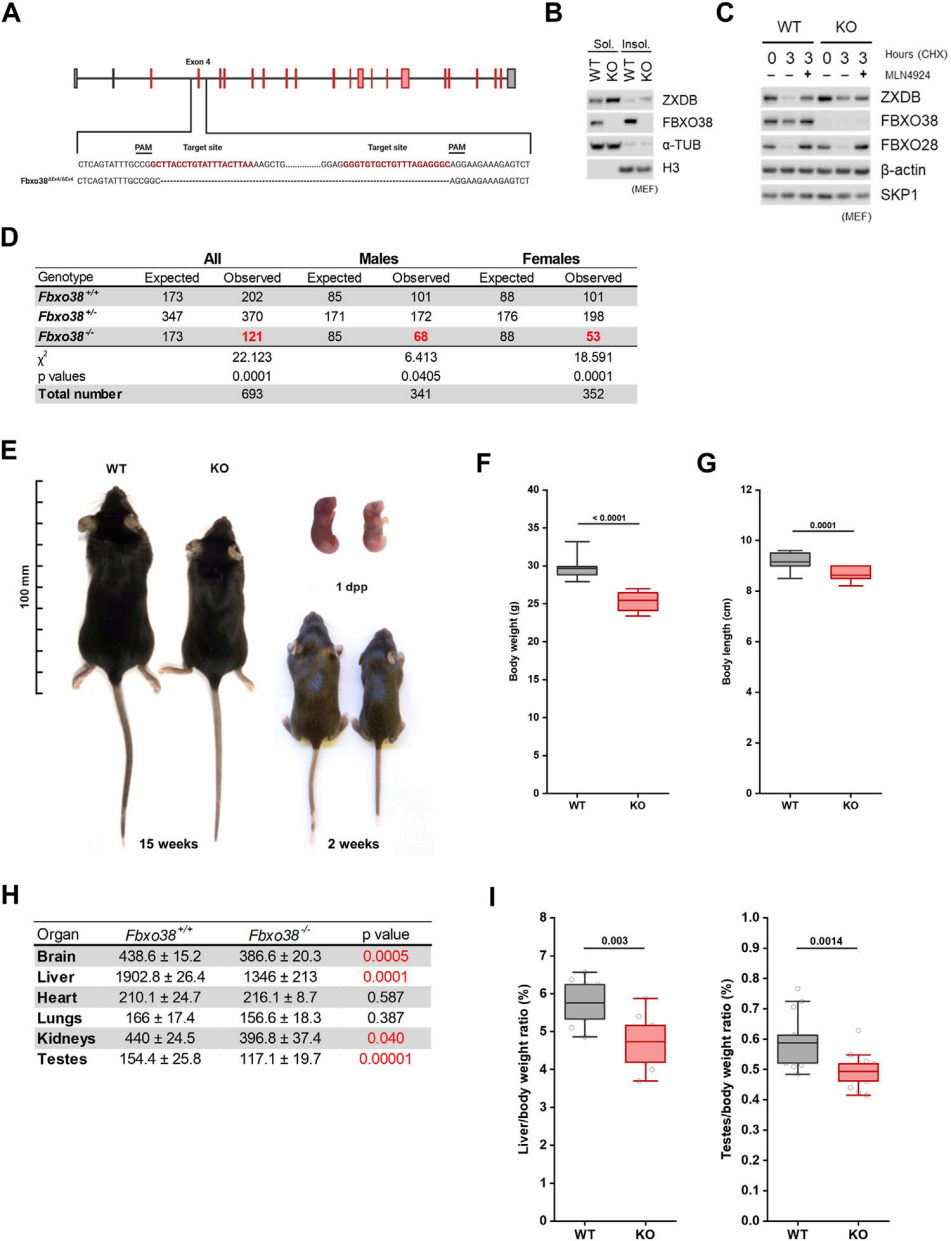
FBXO38 deficiency leads to growth retardation in mouse. **(A)** Scheme illustrating mouse Fbxo38 gene structure and its targeted disruption using a CRISPR/Cas9 genome-editing system. Two single guide RNAs (sgRNAs) were designed to target intron sequences (sequence in red) flanking exon four of the *Fbxo38* gene. Translated exons are depicted in red color. PAM; protospacer adjacent motif, dashed line indicates deleted nucleotides in *Fbxo38* ^ΔEx4/ΔEx4^ animals. **(B)** SV40 immortalized MEF generated from *Fbxo38* wild-type (WT) and knockout (KO) E13.5 embryos were subjected to lysis. Soluble and insoluble fractions were immunoblotted as indicated; ɑ-tubulin and histone H3 were used as loading and fractionation controls. **(C)** MEF (as in C) were treated with cycloheximide (CHX) for 3 h and pre-treated with MLN4924 for 1 h where indicated. Whole-cell lysates were immunoblotted as indicated. Staining of FBXO28 was used as a control for CUL1-dependent degradation. **(D)** Observed and expected mouse genotypes of weaned pups from heterozygous breedings. The proportion of mice with different genotypes was compared to expected Mendelian ratios by the Chi-square test. Significant deviations from the expected numbers are shown in red. **(E)** Representative images of Fbxo38 WT and KO littermate males of indicated age. Dpp; days postpartum. **(F)** Body weight of adult (17–21 weeks) *Fbxo38* WT and KO males. Age distribution in both groups was the same (*n* = 15). Statistical significance was assessed by an unpaired two-tailed *t*-test. **(G)** Body length of animals as in (E). **(H)** Means ± standard deviations and p-values of organ weights of *Fbxo38* WT and KO males as in (E) are summarized in the table. Statistical significance was assessed by an unpaired two-tailed *t*-test. Statistically significant p-values are highlighted in red (p-value < 0.05). **(I)** Liver (left) and testes (right) weight to body weight ratio of adult *Fbxo38* WT and KO males (liver: *n* = 9; testes: *n* = 14). Individual data points are shown. Horizontal bars show mean values, boxes represent 25th and 75th percentiles. Statistical significance was assessed by an unpaired two-tailed *t*-test.

Ubiquitin substrate receptor FBXO38 is highly conserved in vertebrates, with ∼95% amino acid identity between the mouse and human orthologs (Supplementary Figures S1A,B). Identically to human FBXO38 protein, its mouse ortholog binds ZXDA/B proteins *via* degron located in between their fourth and fifth zinc fingers (Supplementary Figure S1C). To uncover the physiological role of FBXO38, we generated a mouse model with a targeted deletion of exon four of the *Fbxo38* gene (*Fbxo38* ^ΔEx4^). Mutated allele encodes prematurely terminated protein without any functional domain, including the F-box motif (Figure 1A, Supplementary Figure S1D). As expected, lysates from immortalized *Fbxo38* ^ΔEx4/ΔEx4^ (KO) mouse embryonic fibroblasts (MEF) lacked any detectable FBXO38 protein. The effect of FBXO38 inactivation was a stabilization of its substrate ZXDB (Figures 1B,C). While heterozygous *Fbxo38* ^WT/ΔEx4^ (HT) animals were fertile with normal gross morphology, the offspring of heterozygous breeding pairs were not born in the expected (Mendelian) ratio. Compared to wild-type (WT) pups, the frequency of birth of *Fbxo38*-deficient mice was reduced by 40% (32%—males; 52% females) (Figure 1D). Surviving newborn *Fbxo38*-deficient mice were smaller and exhibited reduced body weight compared to their littermates. This growth retardation phenotype began to manifest during embryonic development, as KO pups were already smaller at birth (Figure 1E, Supplementary Figure S1E). The phenotype persisted throughout the lifespan—mature adult *Fbxo38* KO mice exhibited reduced body weight and decreased body length (Figures 1E–G). A detailed analysis showed differences in their organ size. We observed a significant decrease in the weight of the liver, brain, testes, and kidneys of KO males when compared to their WT littermates, although the heart and lungs were not affected (Figure 1H, Supplementary Figure S1F). Differences in liver and testes weight between WT and KO were significant, even when normalized to total body weight (Figure 1I). Moreover, the observed differences in organ size were confirmed in the female cohort (Supplementary Figure S1G).

Altogether, these findings demonstrate that, although *Fbxo38* is not essential for mouse survival, it is important for proper growth and fitness.

### Reproduction and Spermatogenesis Are Impaired in FBXO38-Deficient Animals

**FIGURE 2:**
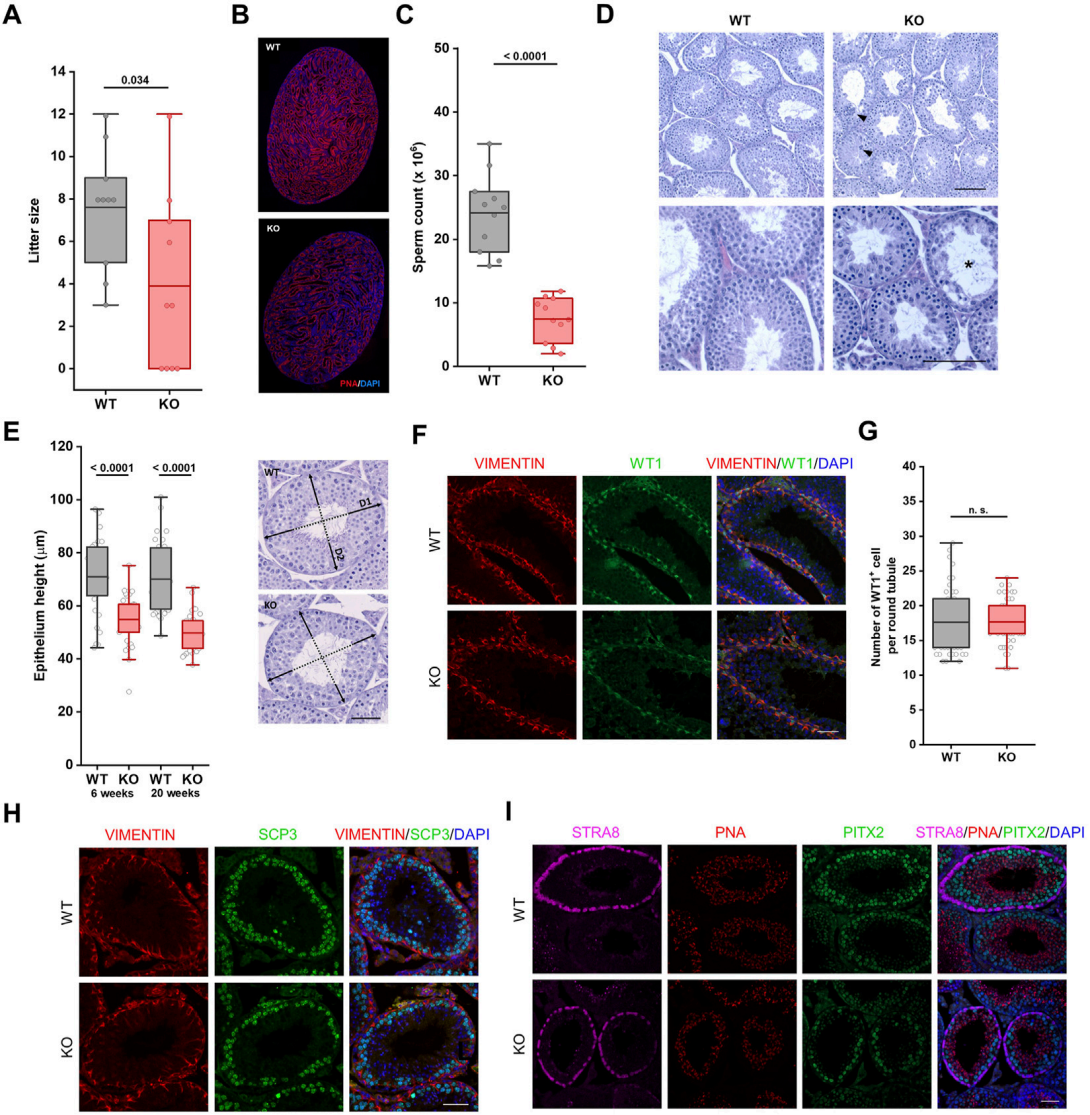
Reproduction and spermatogenesis are impaired in *Fbxo38*-deficient animals. **(A)** Litter size of WT females bred to *Fbxo38* WT or KO males. Litters were recorded for the time period of 18–21 days after monitored vaginal plugs. Number of pups per litter are shown as individual data points, horizontal bars show mean values, boxes represent the 25th and 75th percentiles. Statistical significance was assessed by an unpaired two-tailed *t*-test. **(B)** Composite pictures of sagittal sections of *Fbxo38* WT and KO testes labeled with peanut agglutinin (PNA) lectin and counterstained with DAPI. **(C)** Sperm counts of adult (17–21 weeks) *Fbxo38* WT and KO males with the same age distribution in both groups. Spermatozoa were isolated from both caudae epididymides. Individual data points are shown, horizontal bars show mean values, boxes represent the 25th and 75th percentiles. Statistical significance was assessed by an unpaired two-tailed *t*-test. **(D)** Hematoxylin and eosin-stained testicular sections from 9-week-old *Fbxo38* WT and KO males. Arrowheads point to atypical multinucleated cells, asterisks show disrupted tubules. Scale bar, 100 µm. **(E)** Height of germinal epithelium from young and adult *Fbxo38* WT and KO animals (left) and methodological approach of measurement (right). Average height of germinal layer in two perpendicular axes was measured for each tubule (35 randomly selected round tubules from transversal section per animal). Horizontal bars show mean values, boxes represent the 25th and 75th percentiles. Statistical significance was assessed by an unpaired two-tailed *t*-test. Scale bar, 50 µm. **(F)** Seminiferous tubule sections of young adult (10 weeks) *Fbxo38* WT and KO mice stained for Vimentin and WT1. DNA was visualized with DAPI. Scale bar, 50 µm. **(G)** The number of WT1-positive cells in round seminiferous tubules from sections of *Fbxo38* WT and KO testes (*n* = 2; 24 tubules per animal). Horizontal bars show mean values, boxes represent the 25th and 75th percentiles. Statistical significance was assessed by an unpaired two-tailed *t*-test. **(H)** Seminiferous tubule sections of young adult (10 weeks) *Fbxo38* WT and KO mice stained for Vimentin and SCP3. DNA was visualized with DAPI. Scale bar, 50 µm. **(I)** Seminiferous tubule sections of 17-week-old *Fbxo38* WT and KO mice co-stained with antibodies against STRA8 and PITX2 and labeled with PNA. DNA was visualized using DAPI. Scale bar, 50 µm.

Due to significant differences in the weight of testes between *Fbxo38* WT and KO animals (Figures 1H,I, Supplementary Figure S1F), we decided to determine whether FBXO38 is critical for male reproduction. We conducted breeding experiments where WT female mice were allowed to mate with KO males. In each cage, one female was housed with one male. Mice had free access to food and water. In the first approach, females were checked everyday and once they produced vaginal plug, males were separated (Supplementary Figure S2A). Examining a small-scale cohort of males (*n* = 5), no defects in the mating behavior of KO males were observed and vaginal plugs appeared during the first week of breeding, with no apparent difference between WT and KO males. However, none of the females bred to KO males became pregnant and gave birth to pups, which was in accordance with International Mouse Phenotyping Consortium (IMPC) data (Dickinson et al., 2016). In contrast to this observation, allowing WT females to mate with KO males without separation up to 3 months revealed that KO males are capable of reproduction (Supplementary Figure S2A). However, their reproductive fitness was impaired with significantly smaller litter size (Figure 2A). Furthermore, smaller testes of KO animals correlated with lower spermatid and spermatozoa production (Figure 2B) and a significant decrease in epididymal sperm count (Figure 2C). Despite fewer mature sperms in cauda, the epididymis had a typical gross morphology (Supplementary Figure S2B).

Both young and mature adult KO males showed morphological anomalies in the testes. A histological examination revealed disorganized seminiferous tubules with reduced cellularity at spermatocyte, spermatid, and sperm levels in FBXO38-deficient males (Figure 2D, Supplementary Figure S2C). The disorganized structure of KO seminiferous tubules did not allow us to determine the exact stages of spermatogenesis. However, the height of tubular epithelium was significantly reduced compared to WT testes (Figure 2E). Interestingly, we detected a dramatic increase in the number of multinucleated cells (Supplementary Figures S2C–E).

The histological observation was confirmed using specific antibodies to mark different spermatogenesis populations (The scheme of the antibodies used and publicly available testicular single-cell expression data analysis can be seen in Supplementary Figures S2F,G) (Hermann et al., 2018). First, Sertoli cells were visualized by co-staining of nuclear factor Wilms’ tumor 1 (WT1) and cytosolic intermediate filament vimentin (Figure 2F). We did not observe any differences in Sertoli cell number (Figure 2G). However, compared to WT animal testes, WT1 staining was weaker in the nuclei of the KO Sertoli cells (Supplementary Figure S2H). Markers of the spermatogonial stem cell compartment, Promyelocytic leukemia zinc finger protein (PLZF), and Spalt-like transcription factor 4 (SALL4), did not reveal any significant differences in the number of these cells and the intensity of the staining (Supplementary Figure S2I). Synaptonemal complex protein 3 (SCP3) staining confirmed the disruption of spermatogenesis progression characterized by a reduced number of meiotic spermatocytes (Figure 2H). Immunofluorescence staining with the Stimulated by retinoic acid 8 (STRA8) antibody, the marker of differentiating spermatogonia and target of retinoic acid signaling, allowed us to recognize the distinct stages of spermatogenesis (stage VII-VIII) (Endo et al., 2015). In tubules positive for STRA8, we observed a decreased number of spermatocytes (detected by staining Pituitary homeobox 2; PITX2) and spermatids (detected by peanut agglutinin; PNA), supporting the observation of defects in spermatogenesis. Of note, we observed a weaker signal of STRA8 staining but an undisturbed number of STRA8 positive cells, suggesting a potential disturbance in retinoic acid signaling (Figure 2I).

### The Timing of the First Wave of Spermatogenesis Depends on FBXO38

**FIGURE 3:**
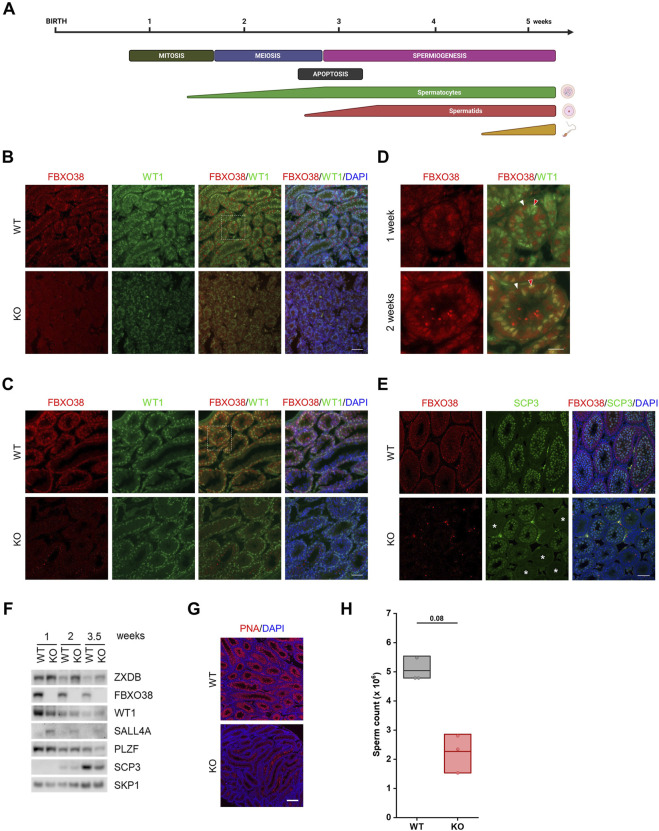
The timing of the first wave of spermatogenesis depends on FBXO38. **(A)** Schematic illustration of major events in the first wave of spermatogenesis. The timeline in the upper panel indicates postnatal age in weeks. The middle panel shows the occurrence of the individual processes in spermatogenesis and the lower panel indicates the time course of the formation of spermatogenic differentiation stages. **(B,C)** Testis sections of infant *Fbxo38* wild-type (WT) and knockout (KO) littermate males at the age of 1 (B) and 2 weeks (C) postpartum stained for FBXO38 and WT1. DNA was visualized with DAPI. Scale bar, 50 µm. **(D)** Details of FBXO38 and WT1 localization in seminiferous tubule sections from WT mice at the age of 1 and 2 weeks from **(B,C)**. White arrowheads show spermatogonia, red arrowheads point to Sertoli cells. Scale bar, 20 µm. **(E)** Co-immunofluorescence staining for FBXO38 and SCP3 of testis sections from *Fbxo38* WT and KO mice at the age of 2 weeks. DAPI was used to stain DNA. Asterisks show tubules with negative SCP3 staining. Scale bar, 50 µm. **(F)** Western blot analysis of whole testes protein lysates from *Fbxo38* WT and KO littermate males at the indicated age. Testes without tunica albuginea were homogenized and lysed in the presence of Benzonase nuclease and lysates were immunoblotted as indicated **(G)** Testis sections of *Fbxo38* WT and KO littermate males at the age of 4.5 weeks labeled with peanut agglutinin (PNA) lectin and counterstained with DAPI. Scale bar, 200 µm. **(H)** Sperm counts of juvenile *Fbxo38* WT and KO mice at the age of 6 weeks. Spermatozoa were isolated from the caudae epididymides. Individual data points are shown, horizontal bars show mean values, boxes represent the 25th and 75th percentiles. Statistical significance was assessed by the Mann-Whitney *U* test.

Decreased sperm counts in adult KO males indicate the importance of FBXO38 in spermatogenesis. Adult mouse spermatogenesis depends on the progression of its first wave characterized by the synchronous initiation of cell divisions, germ cell apoptosis, and, finally, production of the first spermatozoa by 6 weeks of age (Figure 3A). To determine whether this process is affected by FBXO38 deficiency, we first focused on identifying the cellular population expressing FBXO38 protein. We isolated juvenile testes and noticed that, as with adult animals, KO mice testes were smaller in size (example in Supplementary Figure S3D). Initially, FBXO38 protein was expressed predominantly by spermatogonia. However, the staining of testes from two-week-old animals, revealed that its expression broadened also to Sertoli cells, a population of somatic cells essential for testis formation and spermatogenesis (co-stained with WT1) (Figures 3B,C, and detail in 3D). Moreover, immunofluorescence staining showed that many individual tubules lacking FBXO38, did not enter the meiotic stage, suggesting asynchronicity of the first wave of KO animals (Figure 3E). Similar results were obtained by analysis of total testicular protein, which showed a significant decrease in the SCP3 meiotic marker expression in KO testis lysates. As expected, ZXDB was stabilized in KO testes, while WT1 was decreased in accordance with the immunofluorescence staining. Interestingly, we observed a higher expression of SALL4A, a marker of differentiating spermatogonia (Figure 3F). Although this transcription factor contains two zinc-finger linkers bearing the “signature” of a ZXDB-like FBXO38-dependent degron (Supplementary Figure S3A), we could not detect the interaction between FBXO38 and SALL4A (Supplementary Figure S3B).

To support our observation indicating that KO males have a delay in the first wave of spermatogenesis, we visualized spermatids in 4.5-week-old testes. In contrast to WT tubules, which showed the synchronous emergence of spermatids at this age, KO tubules mostly lacked any positivity, suggesting an apparent arrest or delay in the final stages of spermatogenesis. (Figure 3G). Finally, we measured sperm counts of six-week-old males. Although the significance of lower sperm counts from KO males is not strong (due to the smaller sample size; *n* = 3), there is a clear trend showing that the first wave of spermatogenesis is weaker compared to WT littermates (Figure 3H). Additionally, a detailed analysis of the sperm shows no difference in sperm structure or motility (Supplementary Figure S3C + Supplementary Movies S1-WT, S2-KO). Furthermore, we did not observe any differences in the numbers of spermatogonia (Supplementary Figure S3D) or apoptotic cells detected by TUNEL assay staining (Supplementary Figure S3E).

In summary, the analysis of prepubertal testes showed that the localization of FBXO38 expression changes in the early stages of spermatogenesis. Furthermore, our results revealed that the absence of FBXO38 led to a delay in meiotic entry and, consequently, to a lower sperm count.

### FBXO38 Controls ZXDB Protein and the Centromeric Chromatin in Adult Sertoli Cells

**FIGURE 4:**
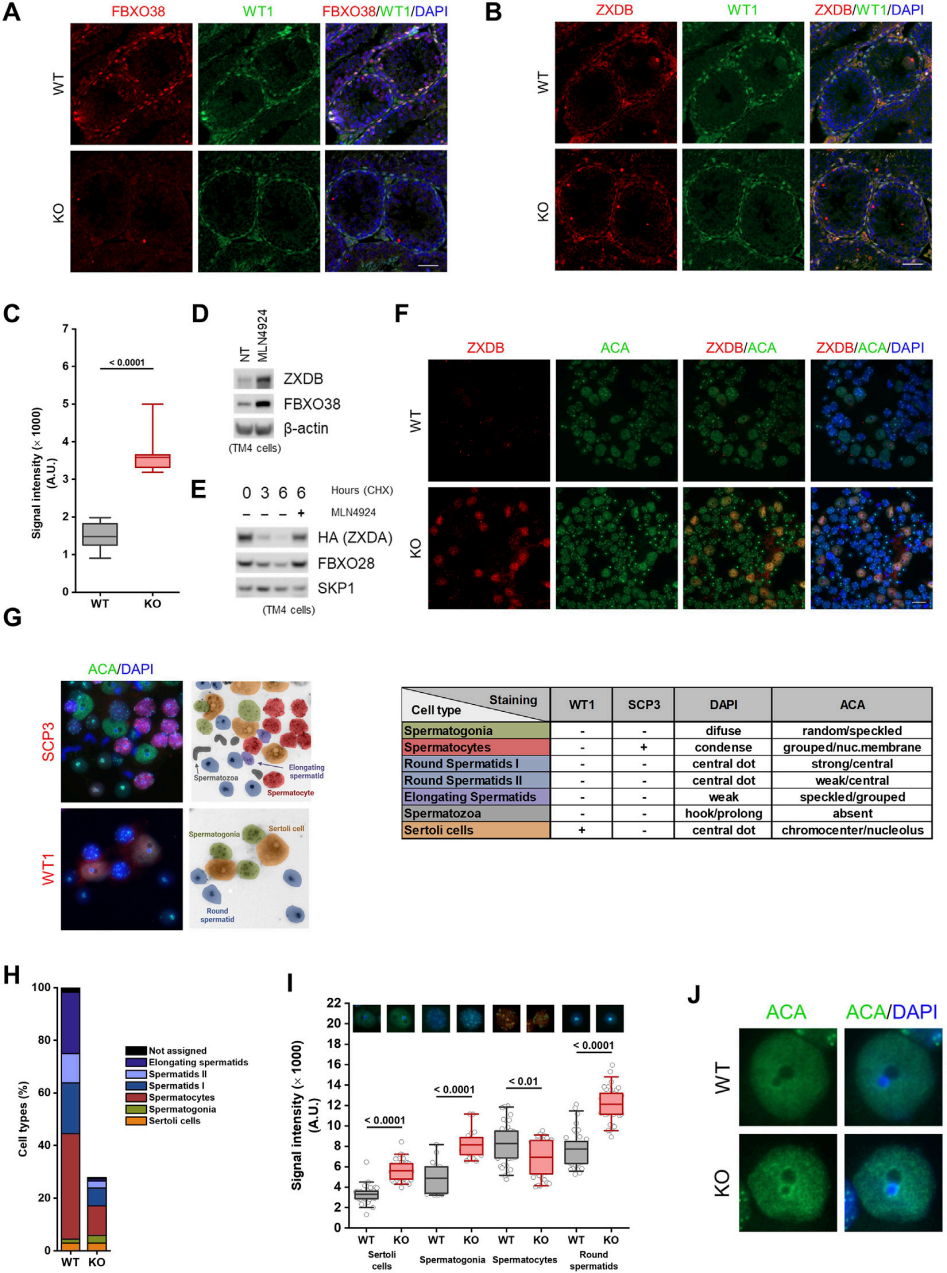
FBXO38 controls ZXDB protein and the centromeric chromatin in adult Sertoli cells. **(A,B)** Seminiferous tubule sections of young adult (10 weeks old) *Fbxo38* wild-type (WT) and knockout (KO) mice co-stained for WT1 and FBXO38 (A) or ZXDB (B). DNA was visualized with DAPI. Scale bar, 50 µm. **(C)** Signal intensity of Sertoli cells from testicular sections of *Fbxo38* WT and KO stained for ZXDB as in (B). The maximum normalized intensity of cells (*n* = 2; 15 Sertoli cells per animal) was measured. Horizontal bars show mean values, boxes represent the 25th and 75th percentiles. Statistical significance was assessed by an unpaired two-tailed *t*-test. **(D)** TM4 cells were either non-treated or treated with MLN4924 for 22 h. Whole-cell lysates were immunoblotted as indicated. **(E)** TM4 stably expressing HA-tagged ZXDA were subjected to cycloheximide chase. Where indicated, cells were pre-treated with MLN4924 for 1 h. Whole-cell lysates were immunoblotted as indicated. **(F)** Seminiferous tubule squash from adult *Fbxo38* WT and KO mice stained with ZXDB antibody and anti-centromere antibodies (ACA). DNA was visualized with DAPI. Scale bar, 20 µm. **(G)** Representative images of testicular cell types (from KO animals) and staining for their characteristic markers used to identify the cell populations in a seminiferous tubule squash. The middle scheme illustrates the position and cell type shown in the left pictures. The table on the right depicts combination of the markers and features used for identification. **(H)** Composition of cell types in a seminiferous tubule squash as in (D). Each fraction is displayed as a percentage from the WT whole-squash count. Sertoli cell count was used for normalization between KO and WT animals. **(I)** The signal intensity of ACA staining in indicated cell types from testicular sections of *Fbxo38* WT and KO mice as in (D). Maximum normalized intensity was measured. Individual data points from two independent experiments are shown. Horizontal bars show mean values, boxes represent the 25th and 75th percentiles. Statistical significance was assessed by an unpaired two-tailed *t*-test. **(J)** Representative magnified images of Sertoli cells from a seminiferous tubule squash from *Fbxo38* WT and KO mice stained with ACA.

Since we established that FBXO38 protein expression shifts from spermatogonia to Sertoli cells during testicular maturation, we decided to determine its expression in adult animals. An analysis of publicly available scRNA-seq data revealed that *Fbxo38* is preferentially expressed in spermatogonia, spermatocytes, Sertoli, and Leydig cells, while *Zxdb* is expressed only in Sertoli cells (Supplementary Figures S4A,B) (Hermann et al., 2018; Ernst et al., 2019). Testicular tubules were first stained with the FBXO38-specific antibody to confirm these data. As with older pups, Sertoli cells showed the highest FBXO38 protein positivity, while spermatogonial cells, but no other stages, expressed FBXO38 slightly above the background level (Figure 4A). In agreement with our previous data, the FBXO38 substrate ZXDB was significantly stabilized in *Fbxo38* KO Sertoli cells in which both genes are co-expressed (Figures 4B,C). Moreover, endogenous ZXDB protein level increased in mouse Sertoli-derived cell line TM4 treated with Neddylation inhibitor MLN4924 which prevents all cullin-dependent ubiquitination (Figure 4D). Additionally, exogenous ZXDA expressed in TM4 cells was quickly degraded upon ribosomal inhibition in the cullin-dependent manner (Figure 4E). Considering our previous observations that ZXDB controls the centromeric chromatin proteins, we optimized their staining in distinct spermatogenic cell types using anti-centromeric antibodies (ACA) and the tubule squash method (Earnshaw and Cooke, 1989; Page et al., 1998; Parra et al., 2003). We confirmed that the ZXDB protein is uniquely stabilized in FBXO38-deficient Sertoli cells (Figure 4F). Analyzing scRNA-seq data revealed that *Cenpa* is expressed strongly by spermatogonia and weakly by Sertoli cells. On the other hand, *Cenpb* is specifically expressed by Sertoli and Leydig cells (Supplementary Figures S4A,C) (Hermann et al., 2018; Ernst et al., 2019). Testicular spreads were co-stained with SCP3 and WT1 markers and ACA to quantify centromeric chromatin signals. This staining allowed us to distinguish spermatogonial cells, Sertoli cells, spermatocytes, round spermatids I, round spermatids II, and elongating spermatids (Figure 4G). We confirmed that KO testes exhibit a lower ratio of spermatocytes and spermatids to Sertoli cells (Figure 4H). *Via* detailed analysis of ACA staining, we showed that the absence of FBXO38 leads to the stabilization of centromeric chromatin proteins, similar to the extent observed in human cancer cell lines (Figure 4I). In Sertoli cells, centromere staining was not concentrated in distinct dots but displayed rather diffuse signals centered around nucleoli-associated chromocenters (Figure 4J). As shown previously, chromocenter association with nucleoli impacts ribosomal gene transcription (Haaf et al., 1990). To assess whether KO Sertoli cells exhibit differentially active nucleoli, we employed staining of B23 (nucleophosmin) protein, previously used as a marker of ribosomal activity (Yung et al., 1985; Maggi et al., 2008). Analysis of B23 fluorescence signal in WT and KO animals showed a significant decrease in mutant Sertoli cells (Supplementary Figures S4D,E).

### FBXO38-Deficient Sertoli Cells Exhibit a Maturation Defect

**FIGURE 5:**
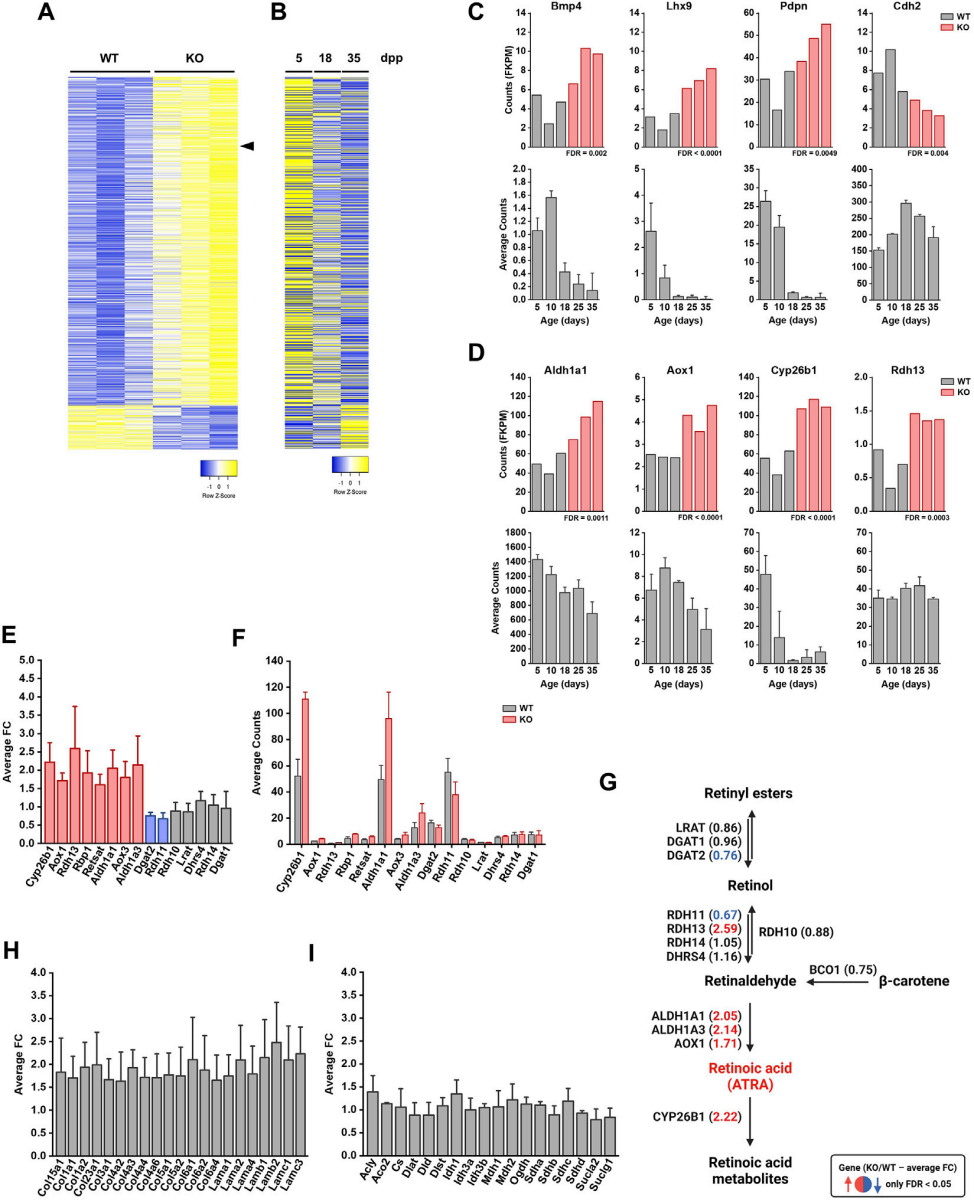
FBXO38-deficient Sertoli cells exhibit a maturation defect. **(A)** Normalized counts (fragments per kilobase million, FKPM) from RNA-seq analysis of *Fbxo38* wild-type (WT) and knockout (KO) young adult (8 weeks old) Sertoli cells were visualized as a heat map showing gene expression normalized to the highest and lowest obtained count. Only 1,170 significantly regulated genes (FDR <0.05) are shown. The arrowhead points to Cyp26b1 gene. **(B)** Publicly available data of gene expressions in Sertoli cells from a different age of juvenile mouse males. The same set of genes as in (A) was visualized as a heat map showing gene expression normalized to the highest and lowest obtained count. Each time point represents the mean value of three different Sertoli cells isolations (GEO accession number GSE59698) (Zimmermann et al., 2015). Publicly available data were normalized using a GREIN application (http://www.ilincs.org/apps/grein/) (Mahi et al., 2019); dpp, days postpartum. **(C)** Upper panel: Expression (FKPM) of four markers of Sertoli cell maturation (Bmp4, Lhx9, Pdpn, and Cdh2) in *Fbxo38* WT (grey) and KO (red) Sertoli cells (dataset as in A). Lower panel: Time course of publicly available gene expression of the same set of genes during Sertoli cell maturation. Each time point represents the mean value obtained from three different Sertoli cell isolations. Publicly available data were obtained and normalized as in (B). **(D)** Upper panel: Expression (FKPM) of four genes involved in retinoic acid metabolism (Aldh1a1, Aox1, Cyp26b1, and Rdh13) in *Fbxo38* WT and KO Sertoli cells (dataset as in A). Lower panel: Time course of publicly available gene expression of the same set of genes during Sertoli cell maturation. Each time point represents the mean value obtained from three different Sertoli cell isolations. Publicly available data were obtained and normalized as in (B). **(E)** An average fold change (KO/WT; *n* = 3) of genes involved in retinoic acid metabolism. The graph represents an excerpt from the dataset shown in (A). Red columns show significantly upregulated genes in KO Sertoli cells (FDR <0.05). The blue columns show significantly downregulated genes in KO Sertoli cells (FDR <0.05). **(F)** An average count (FKPM) of genes involved in retinoic acid metabolism. The graph represents an excerpt from the dataset shown in (A). Each bar represents the mean value (WT—grey; KO—red; *n* = 3). **(G)** Schematic representation of retinoic acid metabolism with depicted fold changes in brackets—as in (D). The red font was used to highlight significantly upregulated genes in KO Sertoli cells (FDR <0.05). The blue font was used to highlight significantly downregulated genes in KO Sertoli cells (FDR <0.05). **(H)** Average fold changes (KO/WT; *n* = 3) of genes encoding laminins and collagens. The graph represents an excerpt from the dataset shown in (A). **(I)** Average fold changes (KO/WT; *n* = 3) of genes involved in the citric acid (Krebs) cycle. The graph represents an excerpt from the dataset shown in (A).

To investigate the molecular consequences of the loss of FBXO38 in Sertoli cells in greater detail, we isolated the cells from 8-week-old WT and KO testes using fluorescence-activated cell sorting (FACS) (Lakpour et al., 2017) (Supplementary Figures S5A,B). Next, we performed a transcriptomic analysis using RNA-seq methodology. A comparison of transcriptomes from *Fbxo38* WT and KO animals revealed significant transcriptional changes (Figure 5A; detail in Supplementary Figures S5C). One of the most significantly upregulated genes in KO Sertoli cells was *Cyp26b1* which encodes an inhibitor of retinoic acid metabolism (Supplementary Figures S5C, marked in Figure 5A with arrowhead). *Cyp26b1* is the marker of immature Sertoli cells, and its transcriptional level declines postnatally (Edelsztein et al., 2020). To assess whether FBXO38 deficiency results in a maturation defect in Sertoli cells, we profiled publicly available data from Sertoli cells isolated during the maturation period. Side-by-side correlation between genes significantly up- or downregulated in FBXO38-deficient Sertoli cells shows an apparent shift toward an immature profile in KO cells (Figures 5A,B). This defect is highlighted in the expression level of genes associated with the maturation of Sertoli cells (Figure 5C) (Baumal et al., 1989; Birk et al., 2000; Pellegrini et al., 2003; Jiang et al., 2015).

An important process associated with maturation is the activation of retinoic acid signaling. As mentioned above, CYP26B1 acts as an inhibitor of retinoic acid signaling by actively metabolizing all-trans retinoic acid (ATRA) into its inactive forms. A short ATRA presence is necessary for the coordinated activation of spermatogonial mitotic division. However, *Cyp26b1* was upregulated in FBXO38-deficient cells, suggesting a disruption in the timed activation of this morphogen signaling pathway (Figure 5D). To obtain a complete picture of this pathway, we profiled an entire set of genes associated with retinoic acid signaling, and this analysis showed the scale of disruption of ATRA metabolism in KO testes (Figures 5D–F). Significant changes are highlighted in the illustrative scheme of this signaling pathway (Figure 5G). Furthermore, the gene ontology (GO) and over-representation analysis (ORA) revealed substantial changes in other signaling pathways and gene clusters (Supplementary Figures S5D,E). We identified a significant upregulation in the genes encoding extracellular matrix components of the basal lamina surrounding the seminiferous tubules (Figure 5H). Moreover, several targets of the Hippo signaling pathway, a sensor of intercellular and cell-to-extracellular matrix interactions, were deregulated (Supplementary Figures S5F).

Other metabolic and signaling pathways were also profiled to characterize the activity of KO Sertoli cells. While genes involved in the TCA (or Krebs) cycle were not altered between WT and KO cells (Figure 5I, Supplementary Figures S5), components and transcriptional targets of the Wnt and Hedgehog signaling pathways were upregulated (Supplementary Figures S5G). To obtain a complete picture of the paracrine activity of mutant Sertoli cells, we profiled the expression of secreted ligands known to be active and vital in Sertoli cell-dependent regulation of spermatogenesis. In addition to previously mentioned factors, such as *Wnt5* and *Wnt6*, and Bone morphogenetic protein 4 (*Bmp4*), changes in expression of an array of these secreted ligands, including Inhibin A, a negative spermatogenesis inhibitor secreted by Sertoli cells, were observed (Supplementary Figures S5H). Importantly, genes upregulated by follicle-stimulating hormone (FSH) or testosterone signaling were not altered in mutant Sertoli cells, suggesting that their differential gene expression profile is not the result of an endocrine imbalance (Supplementary Figures S5F) (Chaudhary and Skinner, 1999; Hu et al., 2010).

In summary, we have shown that the absence of FBXO38 disrupts maturation processes in Sertoli cells, a phenotype correlating with pathological changes observed in seminiferous tubules of FBXO38-deficient mice.

## Discussion

In our study focused on the role of FBXO38 *in vivo*, we demonstrate that loss of FBXO38 results in reduced prenatal and postnatal growth of several organs and tissues, especially male reproductive organs. To determine how FBXO38 acts on the cellular level, we focused on postnatal testicular development, as male reproduction and the testicular epithelium are severely affected in FBXO38-deficient mice.

In early postnatal development, we show that FBXO38 is expressed predominantly in spermatogonial stem cells. These cells start to divide and differentiate during the first wave of spermatogenesis, giving rise to a group of progenitor cells and, consequently, spermatocytes and spermatids. Although we did not observe a general decrease in spermatogonia number in individual *Fbxo38* KO tubules compared to WT, the smaller size of juvenile KO testes implied the their overall number would be lower. Importantly, FBXO38 seems to be predominantly expressed by Sertoli cells in adult testis, although we cannot rule out that FBXO38 expression in adult spermatogonia is below the immunodetection limit and they might still be affected by its loss. Moreover, we observed that the SALL4A protein, suppressor of spermatogonia differentiation (Yamaguchi et al., 2015), is upregulated during the first wave of spermatogenesis in FBXO38-deficient testes, suggesting either deregulation of its gene expression or its aberrant protein degradation. Such aberrant SALL4A expression could alter the dynamic of spermatocyte emergence, as we observed in juvenile testes of FBXO38-deficient mice. Moreover, a comparable effect of aberrant *Sall4* expression on stem cells differentiation was observed in the hematopoietic system (Milanovich et al., 2015). Interestingly, *Sall4a* expression is controlled by all-trans retinoic acid (ATRA) signaling (Gely-Pernot et al., 2015), which is crucial for the regulation of spermatogenesis (Gewiss et al., 2020). ATRA is released from Sertoli cells, and it activates a pro-mitotic program in spermatogonia (Endo et al., 2017). In differentiated spermatogonia, ATRA promotes the expression of an essential gatekeeper of meiotic initiation *Stra8* (Koubova et al., 2006). As it is activated only during a distinct spermatogenic tubule stage, STRA8 is commonly used as a marker for ATRA activity and to determine a specific stage (VII-VIII) in tubule development (Gewiss et al., 2020). By using this staging method, we revealed that *Fbxo38* KO animals consistently show weaker STRA8 staining and, at the same time, a significant reduction in the SCP3^+^ spermatocyte population. Taken together, these observations strongly suggest that FBXO38-deficiency results in defective differentiation of spermatogonia.

Testes isolated from FBXO38-deficient mice were substantially smaller when compared to WT. Since we found that FBXO38 is predominantly expressed in Sertoli cells in adult testes, the major determinant of testis size (O'Donnell et al., 2022), we decided to focus on FBXO38 function in this somatic cell type. By detailed transcriptomic analysis of WT and KO Sertoli cells, we discovered that RA metabolism was one of the most affected pathways. *Aldh1a1* and *Aldh1a3* (otherwise known as *Raldh1/2*), both upregulated in KO Sertoli cells, are the genes responsible for the metabolic conversion of retinaldehyde and ATRA. They were shown to be necessary for spermatogonia differentiation, and their overexpression should lead to an increased amount of ATRA (Raverdeau et al., 2012). As the activation of RA-dependent genes has to be under strict spatiotemporal control, ATRA-mediated signaling leads to transcriptional activation of the *CYP26B1* gene. This enzyme is required for the accelerated clearance of ATRA from cells, and its expression decreases with the onset of puberty (Li et al., 2009). It is noteworthy that *Cyp26b1* is one of the most upregulated genes in *Fbxo38* KO Sertoli cells. Significantly, a high and precise level of Sertoli cell delivered RA is necessary for spermatogonia mitotic entry (Gewiss et al., 2020) and, accordingly, deletion of the *Cyp26b1* gene or its pharmacological inhibition leads to the premature entry of germ cells into meiosis (Bowles et al., 2006; Koubova et al., 2006). Therefore, we hypothesize that FBXO38 deficiency could disrupt ATRA production control and, thus, inappropriate spermatogonial stimulation. In addition to RA signaling, we observed upregulation of several signaling pathways, such as Wnt, Hedgehog, and Hippo. Moreover, an aberrant expression of the extracellular matrix components could impair spermatogonia activation (Siu and Cheng, 2004). Thus, the significantly different transcriptional landscape of KO Sertoli cells results in spermatogenesis defects in adult FBXO38-deficient animals. Analysis of this profile showed a striking resemblance to the gene expression signature of immature Sertoli cells. However, we did not observe any significant structural differences in mutant Sertoli cells, although the nuclei of adult KO cells seemed smaller in volume, which is one of the characteristics of their immaturity as shown in different mammals (Auharek and de França, 2010; Avelar et al., 2010). Nonetheless, Sertoli cell immaturity is a broad term, and the nucleus shape could be a result of complex changes in seminiferous tubule architecture and its cellular composition. To this point, further studies are needed to show the impact of altered gene expression of KO Sertoli cells on their biology and whether this altered gene expression is the result of maturation arrest or dedifferentiation process.

As with FBXO38, its substrate ZXDB zinc finger protein expression is restricted to Sertoli cells, where it is significantly stabilized in *Fbxo38* KO animals. We have recently discovered that ZXDB acts as a positive regulator of centromeric protein CENP-B. Interestingly, as in the case of *Fbxo38*, *Cenpb* KO mice are growth retarded and have reduced testicular weight (Hudson et al., 1998). In accordance with our data from cancer cell lines, we observed an increased centromeric signal in KO Sertoli cells. Sertoli cells do not have distinguished centromeric signals but rather a diffuse one surrounding chromocenters associated with the nucleolus. The number of chromocenters and the localization and genomic regions associated with them undergo dynamic control during differentiation, as described in different systems, including hematopoietic and Sertoli cells (Krzanowska and Bilinska, 2000; Wijchers et al., 2015). It was shown that the forced location of gene loci to chromocenters leads to their heterochromatinization and subsequent transcriptional silencing (Francastel et al., 1999; Brown et al., 2001). The previous report suggests a correlation between the localization of centromeres to chromocenters and rRNA expression in nucleoli (Haaf et al., 1990). Although we did not specifically measure rRNA production in FBXO38-deficient cells, we observed a significant decrease in their nucleophosmin signal. Notably, the levels of nucleophosmin were previously used to indirectly measure rRNA production in nucleoli (Yung et al., 1985; Maggi et al., 2008). The activity of rRNA genes has an enormous effect on cell function and its differentiation potential. For example, in oocytes, distributions of pericentromeric chromatin around nucleoli (speckled or ring-type) distinguish not only nucleoli activity but also the pluripotent potential of fertilized oocytes. Like the one observed in Sertoli cells, ring-type distribution was associated with silenced rRNA genes and full pluripotency potential (Bonnet-Garnier et al., 2012; Kresoja-Rakic and Santoro, 2019). Interestingly, Sertoli cells are giant cells with an abundant rough endoplasmic reticulum. We hypothesized that their function would require higher production of ribosomes, as shown in other large cell types, including neuronal ones (Lipshultz et al., 1982). Moreover, other factors regulating centromere and pericentromeric chromatin have already been shown to impact adult and fetal testes. Alpha-thalassemia/mental retardation, X-linked (ATRX), which controls the establishment of pericentromeric heterochromatin regions, is essential for Sertoli cell proliferation and survival. Mice deficient in *Atrx* have reduced tubule expansion, ultimately impacting the testicular size and its architecture in adulthood (Bagheri-Fam et al., 2011).

In summary, we have described the impact of FBXO38 loss on murine development. This understudied ubiquitin ligase acts as an important factor in the control of organ size, including the male reproductive system. Specifically, we show that FBXO38 is functionally expressed in Sertoli cells and is required for transcriptional control of their maturation and spermatogonia stimulation.

## Materials and Methods

### Mouse Model

Fbxo38 KO mice (∆exon 4) were generated in a C57BL/6N background using the CRISPR/Cas9 genome-editing system. For this purpose, the Cas9 protein and gene-specific sgRNAs (Integrated DNA Technologies) were used for zygote electroporation using a protocol described previously (Chen et al., 2016). Single guide RNA sequences with the a protospacer adjacent motif (PAM) in underlined bold (3′end) were as follows:

sgRNA target 1: TTA​AGT​AAA​TAC​AGG​TAA​GC**CGG**

sgRNA target 2: GGG​TGT​GCT​GTT​TAG​AGG​GC**AGG**.

The genome editing was confirmed in the founder mouse using primers: 5′-GGC​TGT​TCT​CCT​CTT​TGT​G-3′ and 5′-TCC​TTC​CTC​CTG​CTA​GAC​TCT-3’. The mutant allele was backcrossed for two generations to C57BL/6N mice to obtain homozygous animals. Animals were maintained in a controlled, specific pathogen-free environment at the Animal Facility of the Czech Center for Phenogenomics in Vestec, Institute of Molecular Genetics of the Czech Academy of Sciences, Czech Republic. All animal experiments were approved by the Animal Care and Use Committee of the Institute of Molecular Genetics of the CAS (protocol 115/2016) and were in accordance with the Czech Act No. 246/1992 Coll. and European directive 2010/63/EU.

Organs were collected from the mice after euthanasia. Most experiments were carried out on samples collected from adult mice. To enrich certain cell populations in spermatogenesis and to monitor the first wave of spermatogenesis, we used infant and juvenile littermate mice. The age and number of the mice used in this study is indicated in the figure legends. For mouse embryonic fibroblasts (MEFs) preparation, heterozygous animals were bred until females produced vaginal plugs. At the embryonic day (E) 13.5, females were euthanized and embryos were dissected. For examination of female reproductive organs, females from the same litter were housed in the same cages until dissection, in order to avoid differences in the estrous cycle.

### Sperm Count

To evaluate epididymal sperm counts, both caudae epididymides were dissected, placed into 1 ml of PBS, minced, and shaken to allow the sperms to swim out. After 5 min of incubation, suspensions were diluted for quantitative assessments, and individual counts were determined using the Bürker chamber. For sperm morphology and motility assessment, the sperms were released from cauda and incubated in a medium containing methyl-β-cyclodextrin (0.75 mM) for 30 min at 37°C and observed under Zen Axio Vert. A1 microscope (Zeiss).

### Cell Culture

NIH/3T3 and HEK293T (both American Type Culture Collection, ATCC) cell lines were cultured in Dulbecco′s Modified Eagle′s Medium (DMEM) media supplemented with 10% fetal bovine serum (FBS), and antibiotics (penicillin, streptomycin, and gentamicin). TM4 (ATCC) cell line was cultured in a 1:1 mixture of Ham’S F12 and DMEM supplemented with 5% horse serum and 2.5% FBS and antibiotics. Primary MEFs were isolated from E13.5 mouse embryos. After dissection, head and internal organs were removed, and embryos were minced in 0.05% trypsin/EDTA. After 30 min of incubation, dissociated cells were washed, and cultured DMEM supplemented with 10% fetal bovine serum (FBS), non-essential amino acids, l-glutamine, and antibiotics. For immortalization of MEFs, human cell line HEK293T was co-transfected with pLenti CMV/TO SV40 Small and Large T antigen (Addgene, #22298) along with pCMV-dR8.2 (Addgene, #8455) and pCMV-VSV-G (Addgene, #8454). The cells were transfected using polyethylenimine (PEI MW 25000, Polysciences) and maintained in DMEM supplemented with 10% FBS and antibiotics. The lentivirus containing media was collected 48 h after transfection, mixed with Polybrene (Sigma Aldrich), and used for infecting the primary fibroblasts. TM4 cells stably expressing HA-tagged ZXDA were generated by co-transfection of pBABE-HA-ZXDA along with pCMV-VSV-G (Addgene, #8454) and gag/pol plasmid (Addgene, #14887) into HEK293T cells. The viral containing media was collected 48 h after transfection and used for infecting TM4 cells. Cells were selected in a medium containing puromycin (1 μg/ml). For transient transfection, HEK293T cells were transfected with HA-tagged ZXDA or SALL4 using polyethylenimine (PEI MW 25000, Polysciences) and collected 48 h after transfection. All the cells and cell lines were cultured in a humidified incubator at 37°C with 5% CO2. Cycloheximide (100 μg/ml) or MLN4924 (2 µM) were used where indicated.

### Immunoblotting

For whole testes protein lysates, tunica was mechanically removed, and the tissue was placed in a lysis buffer (150 mM NaCl, 50 mM Tris pH 7.5, 0.4% Triton X-100, 2 mM CaCl2, 2mM MgCl2, 1 mM EDTA with addition of phosphatase and protease inhibitor cocktail (MedChem Express) and Benzonase nuclease (0.125 U/μl; Santa Cruz) and homogenized using TissueLyser (Qiagen). After 30 min of incubation on ice, lysates were mixed with an equal volume of 2% SDS in 50 mM Tris-HCl, pH 8, heated for 5 min at 95°C, and cleared by centrifugation.

For the fractionation of MEF protein lysates, cells were washed with ice-cold PBS and lysed for 10 min in the lysis buffer without Benzonase nuclease, centrifuged for 5 min, at 3,000 rpm, and the soluble fraction was separated. The remaining chromatin fraction was further lysed for 10 min in the same buffer with the addition of Benzonase nuclease (1.25 U/μl), mixed with 2% SDS in 50 mM Tris-HCl, pH 8, to final concentration, 1%, heated for 5 min at 95°C and cleared by centrifugation. Protein concentration was determined by the BCA method (ThermoFisher). Samples were prepared by mixing with Bolt™ LDS Sample Buffer (Thermo Fisher Scientific) supplemented with 10% β-mercaptoethanol (Sigma Aldrich), heated for 5 min at 95°C and then separated using a NuPAGE™ 4–12% gradient Bis-Tris gel (Invitrogen). Afterward, the samples were transferred to a PVDF membrane (Amersham), blocked with 5% milk (ChemCruz), and incubated with indicated antibodies diluted in 3% BSA (PanReac Applichem) in TBS-T overnight at 4°C. The HRP-conjugated secondary antibodies (Cell Signaling) were diluted in 5% milk in TBS-T. The membranes were developed using WesternBright ECL (Advansta) or SuperSignal™ West Femto Maximum Sensitivity Substrate (Thermo Scientific).

### Protein Immunoprecipitation and Affinity Purification

Transfected cells were collected and lysed in the lysis buffer (150 mM NaCl, 50 mM Tris pH 7.5, 0.4% Triton X-100, 2 mM CaCl2, 2 mM MgCl2, 1 mM EDTA, supplemented with phosphatase and protease inhibitors) in the presence of Benzonase nuclease (0.125 U/μl) for 30 min on ice. The lysates precleared by centrifugation were then incubated with Pierce™ anti-HA Magnetic Beads (Thermo Fisher Scientific). After series of washes, immunoprecipitated HA-tagged proteins were eluted with 1x Bolt™ LDS Sample Buffer (Thermo Fisher Scientific). Eluates were then supplemented with β-mercaptoethanol (Sigma Aldrich) and incubated at 95°C for 5 min.

MEF with inducible expression of Strep-FLAG-tagged ZXDA were collected 24 h after doxycycline treatment and lysed in the identical buffer. The lysates precleared by centrifugation were then incubated with Strep-Tactin^®^ Sepharose resin (IBA Lifesciences). Purified Strep-tagged proteins were then eluted by desthiobiotin using Buffer E (IBA Lifesciences) and subsequently prepared for immunoblotting as described above.

### Seminiferous Tubule Squash

Squashed seminiferous tubules were prepared according to the previously published protocol (Page et al., 1998). Isolated testes without tunica albuginea were fixed in 2% formaldehyde in PBS containing 0.05% Triton X-100 for 10 min at room temperature. Isolated tubules were placed on a slide with the fixative, gently minced, and squashed with coverslip. Slides were frozen in liquid nitrogen, the coverslips were removed and slides were three times washed with PBS before immunolabeling.

### Histology and Immunofluorescence Staining

Tissues were fixed in modified Davidson’s fluid for 24 h, dehydrated, and processed in automated tissue processor (Leica) prior to paraffin embedding on paraffin station (Leica). Paraffin sections of 4 µm thickness were deparaffinized, hydrated, and stained with hematoxylin and eosin (H&E) or periodic acid-Schiff (PAS) using Ventana Symphony Slide Stainer (Roche).

Antigen retrieval for immunofluorescence staining was performed in 0.01 M citrate buffer, pH 6, in a steam bath for 18 min. Afterward, slides were permeabilized with 0.2% Triton X-100 in PBS for 10 min and blocked with 5% goat and 5% horse serum for 1 h. The samples were incubated with the indicated primary antibodies in a humidified chamber O/N at 4°C. The signal was visualized using AlexaFluor-conjugated secondary antibodies (Abcam). DAPI was used to counterstain DNA. Slides were mounted with ProLong Gold Antifade Mountant (Invitrogen). For staining of testicular squashes, the slides were washed with PBS and blocked with 3% BSA with 0.1% Triton X-100 in PBS for 45 min. Incubation with the indicated primary antibodies was carried out for 2 h in RT followed by washes. Secondary antibodies incubation, DNA staining and mounting was performed as described above. Images were acquired using Axio Imager Zeiss 2 (EC Plan—Nefluar objectives) and analyzed with ZEN2.3 or ImageJ software. ImageJ macro, used for normalization and quantification of centromeric signal, was developed by IMG microscopy facility and is available upon request. Composite images of H&E or PAS-stained organs were obtained by scanning using Axio Scan. Z1 slide scanner (Zeiss). Composite images of fluorescent-stained testes were obtained on DMi8 inverted microscope (Leica). The apoptotic cells in the tissue sections were determined by a terminal deoxynucleotidyl transferase-mediated dUTP nick-end labeling (TUNEL) assay, using the TACS 2 TdT-DAB *In Situ* Apoptosis Detection Kit (Trevigen) according to the protocol.

### Sertoli Cell RNA Isolation

Dissected testes were decapsulated and processed into a single-cell suspension using a previously described protocol (Bellvé et al., 1977). Briefly, seminiferous tubules were submerged in EKRB medium (120.1 mM NaCl, 4.8mM KCl, 25.2 mM NaHCO3, 1.2 mM KH2PO2, 1.2 mM MgSO4, 1.3 mM CaCl2, 11 mM glucose, 1 mM l-glutamine, 4.4 mM sodium lactate, 6.5 mM sodium pyruvate, 20 mM HEPES, non-essential amino acid and penicillin-streptomycin) with collagenase (0.5 mg/ml) and incubated for 20 min at 32°C. Tubules were disrupted by gently pipetting up and down, followed by filtration through a 100 µm strainer. Cells were sedimented at 300 g for 10 min and resuspended in fresh EKRB medium. To identify the Sertoli cells, the cell suspension was stained with a commercially available antibody against the follicle-stimulating hormone receptor (FSHR) conjugated with phycoerythrin (Bioss, BS-20658R-PE) for 1 h. FSHR-positive Sertoli cells were sorted (Influx; BD Biosciences) into the RLT buffer (Qiagen) supplemented with β-mercaptoethanol. The RNA from the cells was isolated using an RNeasy Micro Kit (Qiagen) according to the manufacturer’s protocol. FACS data were analyzed by FlowJo v10 software.

### RNA-Seq

The quantity and quality of isolated RNA was measured using Qubit (ThermoFisher Scientific) and analyzed by Agilent 2,100 Bioanalyser (Agilent Technologies). Sequencing libraries were prepared with Takara Smarter Stranded Total RNA-seq Kit v2 Pico Input Mammalian (PN 634413). Libraries were sequenced on the Illumina NextSeq^®^ 500 instrument using the 76bp single-end configuration.

For subsequent read processing, a bioinformatics pipeline nf-core/rnaseq version 1.4.2, was used (Ewels, 2019). Individual steps included removing sequencing adaptors and low-quality reads with Trim Galore! (http://www.bioinformatics.babraham.ac.uk/projects/trim_galore/), mapping to reference genome GRCm38 (Ensembl annotation version 98) with HISAT2 and quantifying expression on gene level with featureCounts (Liao et al., 2014; Kim et al., 2015; Cunningham et al., 2019). Per gene mapped counts served as input for differential expression analysis using DESeq2 R Bioconductor package (Love et al., 2014). Prior to the analysis, genes not expressed in at least two samples were discarded. We supplied experimental model assuming sample group as main effect. Resulting per gene expression log2-fold changes were used for differential expression analysis. Genes exhibiting minimal absolute log2-fold change value of log2 (1.5) and statistical significance (FDR <0.05) between compared groups of samples were considered as differentially expressed. As next, gene set over representation analysis was done using gene length bias aware algorithm implemented in goseq R Bioconductor package with KEGG pathways and GO terms gene sets (Young et al., 2010).

For individual genes expression comparison, raw read counts were normalized using fragment counts per kilobase of feature length per million mapped reads (FPKM) based on sum of lengths of all gene exons.

Gene set enrichment analysis (GSEA) was performed using GSEA v.4.1.0 software. Over-Representation Analysis (ORA) was performed using the WEB-based GEne SeT AnaLysis Toolkit (http://www.webgestalt.org/). Publicly available data from the GEO database were normalized using the GREIN application (http://www.ilincs.org/apps/grein/) (Edgar et al., 2002; Mahi et al., 2019).

### Quantification and Statistical Analysis

A heatmap was created with Heatmapper (www.heatmapper.ca) (Babicki et al., 2016); scRNA-seq data were visualized using the Loupe cell browser (https://www.10xgenomics.com/products/loupe-browser). Differences between Fbxo38 WT and KO groups were evaluated by an unpaired two-tailed *t*-test, while a chi-square test was used to assess the mouse offspring ratios. *p*-value < 0.05 was considered significant. To calculate the statistical significance of young male sperm count difference, the non-parametric Mann-Whitney *U* test was performed. For RNA-seq data, *p*-values and FDR were calculated; FDR <0.05 was considered significant. Individual data points and group means are shown in figures. The statistical analyses were performed using OriginPro 2021 (OriginLab). Protein identity and similarity, and MUSCLE alignment was assessed by MegAlign Pro (DNAStar).

The maximum normalized signal intensity was determined by measuring the intensity in a circle corresponding to the diameter of the signal. The average background value (measured in four areas) was subtracted from the obtained signal intensity value. Intensity of cells (the number is specified in each figure) was measured from at least two animals of each genotype. For ACA staining intensity in testicular squashes, the cell types were grouped according to specific features, and at least 20 cells of each cell type were measured in two animals of each genotype in two independent experiments. The number of specifically stained cells was determined by counting these cells from all round tubules obtained from the middle cross-sections and shown as the number of positive cells per round tubule. The number of positive cells from at least two animals of each genotype were measured in two independent experiments. Epithelial height was determined by measuring the diameter of round tubules in two perpendicular axes with subtracting the diameter of the lumen. At least 120 tubules from two animals of each genotype were measured in two independent experiments. All the analyses of microscopy images were performed by ImageJ software or ZEN2.3 (Zeiss). The composition of cell types in a seminiferous tubule squash was determined by counting the cells and grouping them according to specific staining. Fractions were shown as a percentage of the number of whole testis squash from wild-type animals. Sertoli cell count was used as a reference for normalization between genotypes.

Litter size was determined by monitoring the birth within 18–21 days after recording vaginal plug.

## Data Availability

RNA-seq data are deposited at the ArrayExpress database under accession number E-MTAB-11271.
