# Supplementary material for: FBXO38 Ubiquitin Ligase Controls Sertoli Cell Maturation
Source: Front Cell Dev Biol. 2022 Jun 13;10:914053. doi: 10.3389/fcell.2022.914053 (PMC9234700; doi:10.3389/fcell.2022.914053)
Supplement: Supplementary file 3 [file DataSheet1.docx]

Supplementary Material

**FBXO38 Ubiquitin Ligase Controls Sertoli Cell Maturation.**

Nikol Dibus^1,2^, Eliska Zobalova^1^, Mario A.M. Monleon^1^, Vladimir Korinek^3^, Dominik Filipp^4^, Jana Petrusova^4^, Radislav Sedlacek^5^, Petr Kasparek^5^ and Lukas Cermak^1^*

* Corresponding author: Lukas Cermak, [lukas.cermak@img.cas.cz](mailto:lukas.cermak@img.cas.cz)

**Table of Contents**

[Supplementary figures and legends 2](#_Toc104566164)

[Figure S1. FBXO38 deficiency leads to growth retardation in mouse 3](#_Toc104566165)

[Figure S2. Reproduction and spermatogenesis are impaired in Fbxo38-deficient animals 5](#_Toc104566166)

[Figure S3. The timing of the first wave of spermatogenesis depends on FBXO38 7](#_Toc104566167)

[Figure S4. FBXO38 controls ZXDB protein and the centromeric chromatin in adult Sertoli cells 9](#_Toc104566168)

[Figure S5. FBXO38-deficient Sertoli cells exhibit a maturation defect 11](#_Toc104566169)

[Supplementary movies legends 12](#_Toc104566170)

[Movie S1 and S2 (separate files). Sperm motility 12](#_Toc104566171)

[List of reagents 12](#_Toc104566172)

[Chemicals 12](#_Toc104566173)

[Antibodies 13](#_Toc104566174)

[Vectors 14](#_Toc104566175)

[References 14](#_Toc104566176)

# Supplementary figures and legends


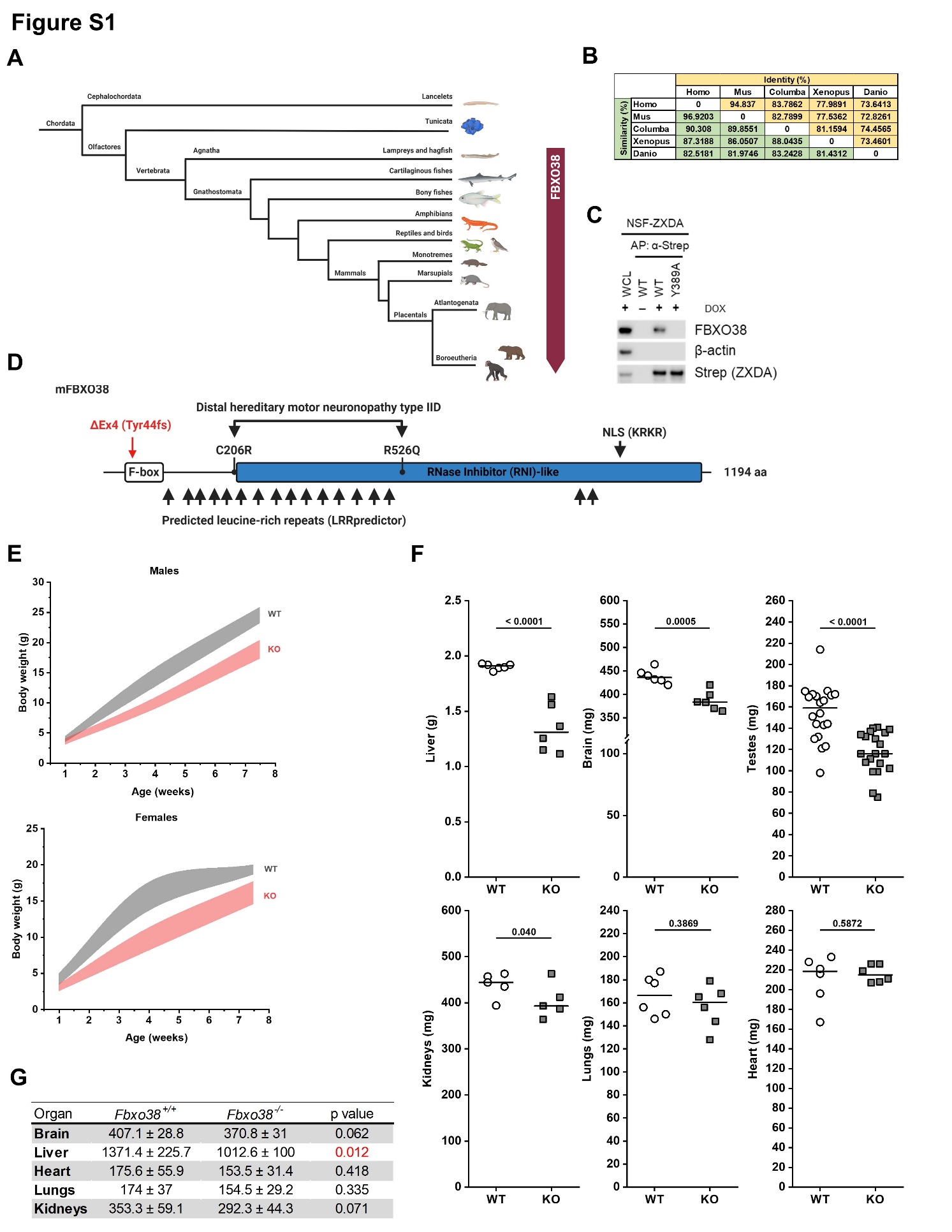


## Figure S1. FBXO38 deficiency leads to growth retardation in mouse

1. A simplified evolutionary scheme illustrating the emergence of *Fbxo38* gene.
2. Table showing the similarity and identity of FBXO38 proteins in different vertebrate species.
3. Mouse embryonic fibroblasts (MEF) with the inducible expression of StrepII-FLAG-tagged ZXDA (SF-ZXDA) and its mutant (Y389A) were treated with doxycycline for 24 hours where indicated. Whole-cell lysates (WCL) were subjected to affinity purification (AP) using Strep-Tactin resin and immunoblotted as indicated. WCL represent 1 % input subjected to AP.
4. A scheme illustrating mouse FBXO38 structural features and disease-associated mutations found in patients with distal hereditary motor neuronopathy IID. The F-Box motif is located in the N-terminus followed by the predicted leucine-rich repeats. Locations of the two mutations (C206R and R526Q) found in patients. The previously confirmed nuclear localization sequence (NLS) is located in the C-terminal part. Red arrow points to frameshift resulting from targeted deletion of exon 4 in mouse model.
5. Growth curves of infant and juvenile *Fbxo38* wild-type (WT) and knockout (KO) males (left) and females (right). Body weights were measured at 1, 4, 7, and 8 weeks of age (n^♂^ ≥ 6; n^♀^ ≥ 3). Curves represent mean values ± standard deviations.
6. Total weight of the liver, brain, testes, both kidneys, lungs and heart of *Fbxo38* WT and KO males. Age distribution in both groups was the same (17-21 weeks). Individual data points and group means are shown. Statistical significance was assessed by an unpaired two-tailed t-test.
7. Means ± standard deviations and p-values of organ weights of adult (25 weeks) *Fbxo38* WT and KO females are summarized in the table. Statistical significance was assessed by an unpaired two-tailed t-test. Statistically significant p-values are highlighted in red (p-value < 0.05).


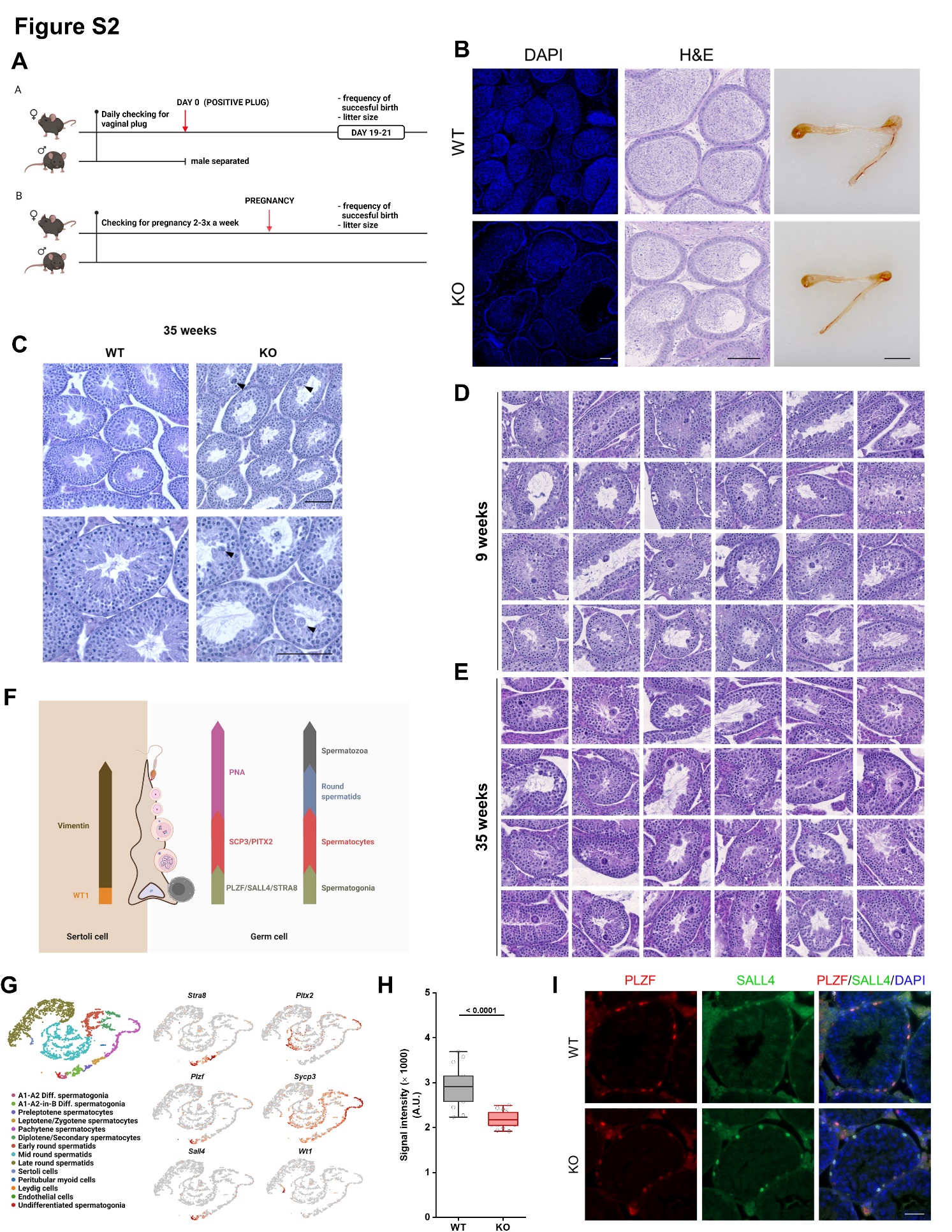


## Figure S2. Reproduction and spermatogenesis are impaired in Fbxo38-deficient animals

1. Schematic representation of strategies used for assessing male fertility.
2. Cauda epididymis of 17-week-old *Fbxo38* wild-type (WT) and knockout (KO) males stained with DAPI (left), or hematoxylin and eosin (H&E; middle). Scale bars, 100 µm. Representative images of *Fbxo38* WT and KO epididymis (right). Scale bar, 500 µm.
3. H&E-stained testicular sections from *Fbxo38* WT and KO males (35 weeks). Arrowheads point to atypical multinucleated cells. Scale bar, 100 µm.
4. – **(E)** Examples of H&E-stained cross-sections of seminiferous tubules bearing multinucleated giant cells from adult *Fbxo38* KO mice – 9 weeks (D), 35 weeks (E).
5. Scheme of antibodies used to visualize different somatic and germ cell populations in testicular cross-sections.
6. tSNE representation of gene expression in adult mouse unselected spermatogenic cells where the color represents the log2-transformed, normalized counts. The enlarged panel on the left shows different spermatogenic populations. The panels on the right show the expression of spermatogonial markers: *Stra8, Plzf,* and *Sall4,* Spermatocytes markers: *Pitx2* and *Sycp3,* and Sertoli cell marker *Wt1*. Publicly available data were uploaded from https://data.mendeley.com/datasets/kxd5f8vpt4/1 and analyzed using the Loupe cell browser (1).
7. The signal intensity of WT1 staining in sections from *Fbxo38* WT and KO testes. The maximum normalized intensity of cells (n=2; 15 Sertoli cells per animal) was measured. Horizontal bars show mean values, boxes represent the 25th and 75th percentiles.
8. Seminiferous tubule sections of adult (15 weeks) *Fbxo38* WT and KO mice stained for PLZF and SALL4. DNA was visualized with DAPI. Scale bar, 50 µm.


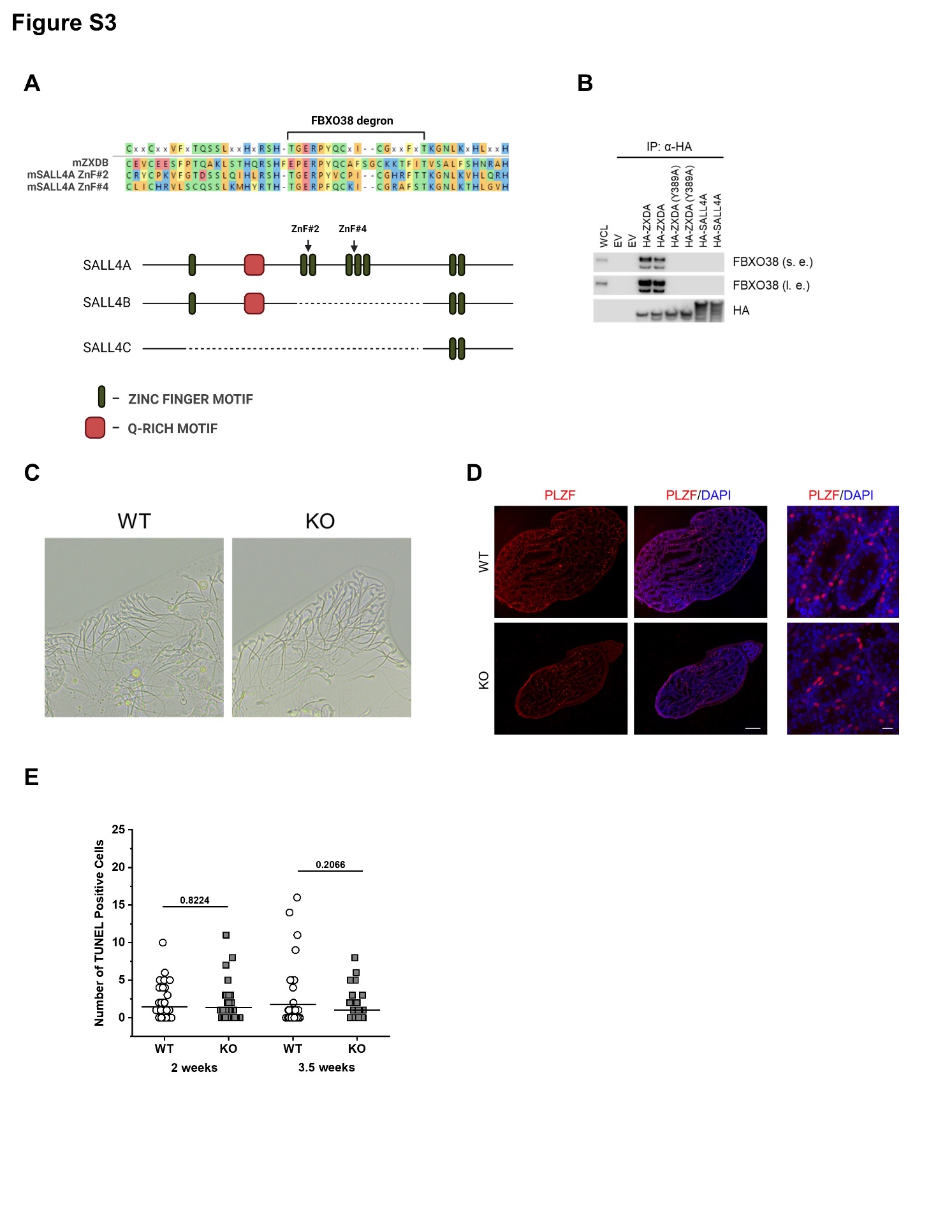


## Figure S3. The timing of the first wave of spermatogenesis depends on FBXO38

1. Analysis of a potential FBXO38-dependent degron in the zinc-finger linkers of SALL4A. The upper panel shows the alignment between the FBXO38-dependent degron in ZXDB and two potential sites in SALL4A. The lower panel shows a comparison of SALL4 isoforms and indications of potential sites location (arrows).
2. HEK293T cells were transfected with an empty vector (EV), HA-tagged ZXDA, ZXDA mutant Y389A, or mouse SALL4A, and treated with MLN4924 inhibitor 6 hours prior to collecting. The cells were subjected to isotonic lysis and immunopurified using anti-HA magnetic beads and immunoblotted as indicated.
3. Representative images of sperm morphology of young adult (6 weeks) *Fbxo38* wild-type (WT) and knockout (KO) mice. Sperms were released from the cauda epididymides. Videos of motile sperms are available as a supplement.
4. Sagittal sections of *Fbxo38* WT and KO testes at the age of 2 weeks stained for PLZF and counterstained with DAPI. Scale bar, 200 µm. Right panel shows the detail of individual tubules. Scale bar, 20 µm.
5. Number of TUNEL-positive cells per tubule (n = 50) in sections of *Fbxo38* WT and KO littermate males at the indicated age. Individual data points are shown, horizontal bars show mean values. Statistical significance was assessed by an unpaired two-tailed t-test.


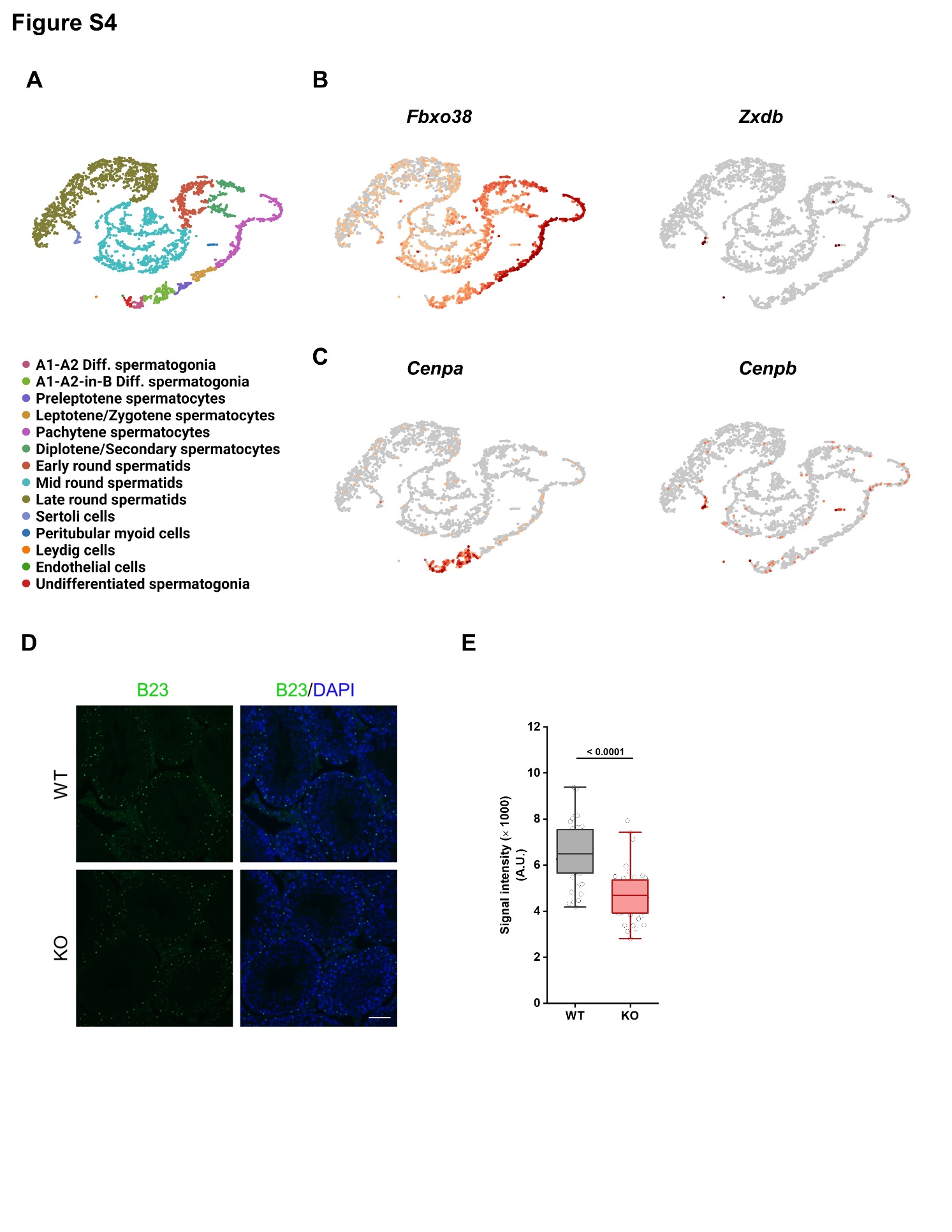


## Figure S4. FBXO38 controls ZXDB protein and the centromeric chromatin in adult Sertoli cells

1. tSNE representation of adult mouse spermatogenic cells where the colors represent the different populations as described in the lower panel. Publicly available data were uploaded from https://data.mendeley.com/datasets/kxd5f8vpt4/1 and analyzed using the Loupe cell browser (1).
2. tSNE representation of *Fbxo38* and *Zxdb* gene expression, where the color represents the log2-transformed, normalized counts. Publicly available data were uploaded and analyzed as in (A).
3. tSNE representation of *Cenpa* and *Cenpb* gene expression analyzed and visualized as in (B).
4. Representative images of tubule sections of adult *Fbxo38* wild-type (WT) and knockout (KO) mice stained for B23. DAPI was used to visualize DNA. Scale bar, 50 µm.
5. Signal intensity of B23 staining in Sertoli cells. Mean normalized intensity was measured in two independent experiments. Individual data points represent a single Sertoli cell measurement (n=2; 40 Sertoli cells per animal). Horizontal bars show mean values, boxes represent the 25th and 75th percentiles. Statistical significance was assessed by an unpaired two-tailed t-test.


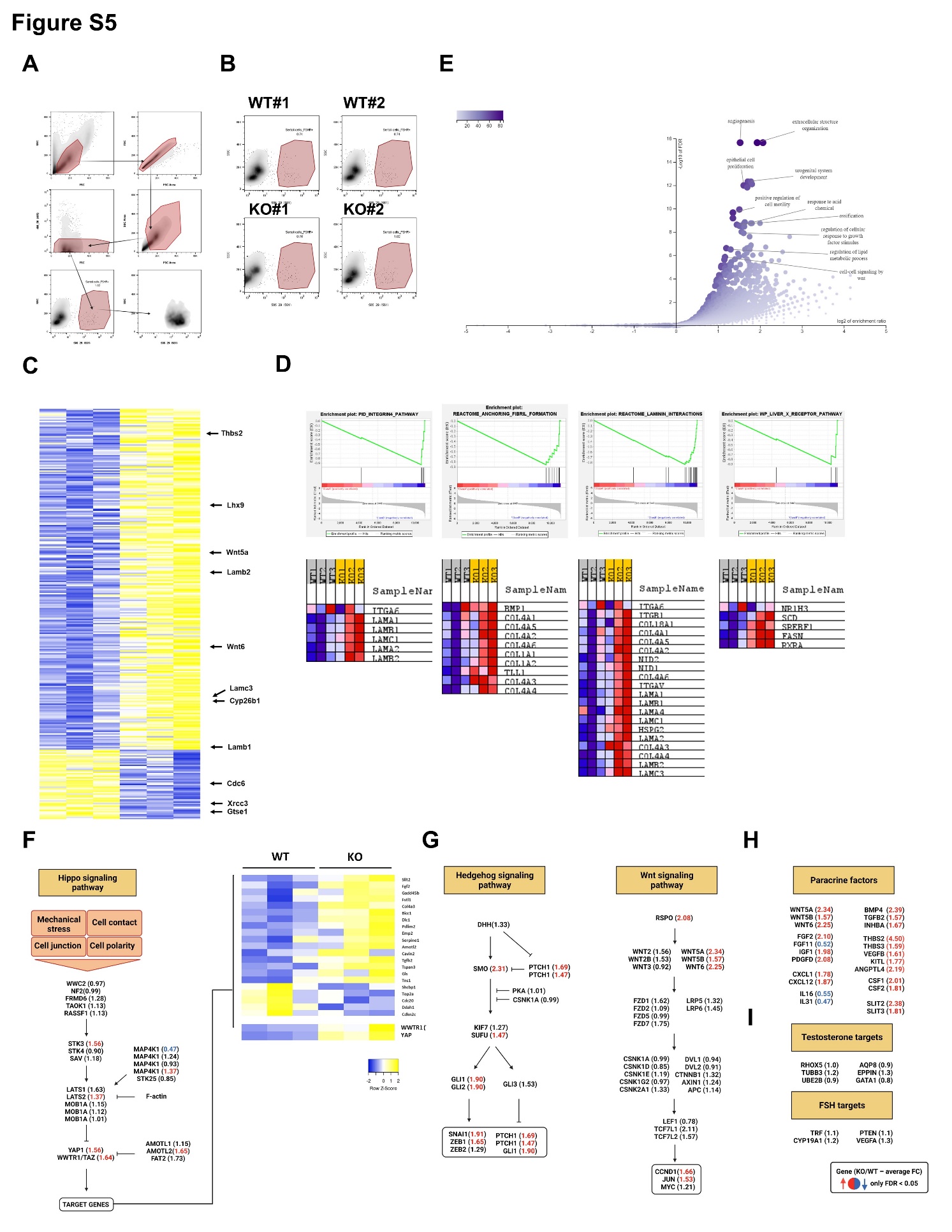


## Figure S5. FBXO38-deficient Sertoli cells exhibit a maturation defect

1. Scheme of the mouse Sertoli cells sorting strategy. Adult Sertoli cells (obtained from 8-week-old animals) were isolated using enzymatic tissue digestion, followed by fluorescence-activated cell sorting with the anti-FSHR antibody.
2. Effectivity of mouse Sertoli cells sorting from *Fbxo38* wild-type (WT) and knockout (KO) testes. Two out of the three isolations are shown.
3. Normalized counts (fragments per kilobase million; FKPM) from the RNA-seq analysis of *Fbxo38* WT and KO young adult (8-week-old animals) Sertoli cells were visualized as a heat map showing gene expression normalized to the highest and lowest obtained count. Only 301 significantly regulated genes (FDR < 0.05; false discovery rate) are shown. Arrows point to individual genes.
4. Gene set enrichment analysis (GSEA) of gene expression data from *Fbxo38* WT and KO Sertoli cells. GSEA enrichment plots of four gene clusters that are enriched in *Fbxo38* KO Sertoli cells are shown. Heat maps of genes from enriched clusters are shown in the lower panel.
5. Over-Representation Analysis (ORA) of gene enrichment was prepared using the WEB-based GEne SeT AnaLysis Toolkit (<http://www.webgestalt.org/>). Positively enriched non-redundant gene clusters are named in the figure.
6. Schematic representation of the Hippo signaling pathway with depicted fold changes in brackets. Data represent an average fold change (*Fbxo38* KO/WT Sertoli cells; n = 3). Significantly upregulated genes in KO Sertoli cells (FDR < 0.05) are in red. The blue font was used to highlight significantly downregulated genes in KO Sertoli cells (FDR < 0.05). Normalized counts (FKPM) of Hippo signaling pathway target genes (YAP conserved signature; GSEA: M2871) (2) from the RNA-seq analysis of *Fbxo38* WT and KO young adult (8 weeks old) Sertoli cells are shown on the right side of the panel. Data were visualized as a heat map showing gene expression normalized to the highest and lowest obtained count. Significantly regulated genes (FDR < 0.05) are depicted in the upper part of the heat map. Canonical transcription factors controlling Hippo-dependent target genes – *Yap* and *Wwtr1* (*Taz*), are shown in the lower part of the heat map.
7. Schematic representation of the Hedgehog and Wnt signaling pathways with depicted fold changes in brackets. Data represent an average fold change (*Fbxo38* KO/WT Sertoli cells; n = 3). Significantly upregulated genes in KO Sertoli cells (FDR < 0.05) are in red. The blue font was used to highlight significantly downregulated genes in KO Sertoli cells (FDR < 0.05).
8. List of paracrine factors with depicted fold changes in brackets. Significantly upregulated genes in KO Sertoli cells (FDR < 0.05) are in red, significantly downregulated genes (FDR < 0.05) are in blue.
9. List of testosterone and FSH target genes with depicted fold changes in brackets. Significantly upregulated genes in KO Sertoli cells (FDR < 0.05) are in red, significantly downregulated genes (FDR < 0.05) are in blue.

# Supplementary movies legends

## Movie S1 and S2 (separate files). Sperm motility

Sperms of young adult (6 weeks) Fbxo38 wild-type (Movie S1) and knockout (Movie S2) mice. The sperms were released from cauda epididymides, incubated in medium containing methyl-β-cyclodextrin (0.75mM) for 30 minutes at 37 °C, and observed under Zen Axio Vert.A1 microscope (Zeiss).

# List of reagents

## Chemicals

| **Name** | **Company** | **CAT#** |
| --- | --- | --- |
| 2-mercaptoethanol | Sigma | M6250 |
| 4',6-diamidin-2-fenylindol (DAPI) | Sigma | D9542 |
| Benzonase | Santa Cruz | sc-391121 |
| Cycloheximide | Sigma Aldrich | C7698 |
| Dithiothreitol | Sigma | 10197777001 |
| Doxycycline hyclate | Sigma Aldrich | D9891 |
| Eosin Y | VWR Qpath | 10047101 |
| Harris' Hematoxylin | VWR Qpath | 10047107 |
| Methanol | Penta | 67-56-1 |
| Paraformaldehyde | Electron Microscopy Sciences | 15710 |
| Pevonedistat (MLN4924) | Medchemexpresss | HY-70062 |
| Polybrene | Sigma | 107689 |
| Polyethyleneimine (MW 25 000) | Polysciences | 23966 |
| ProLong Gold Antifade Mountant | Thermo Fisher Scientific | P36934 |
| Protease Inhibitors Mini Tablets | Pierce | A32955 |
| Proteinase K | Sigma | P2308 |
| Puromycin | Sigma Aldrich | P8833 |
| Sodium dodecyl sulfate (SDS) | Sigma | 71736 |
| Sodium fluoride | Sigma | S7920 |
| Sodium orthovanadate | Sigma | 450243 |
| TACS 2 TdT-DAB (TUNEL assay) | Trevigen | 4810-30-K |

## Antibodies

| **Primary antibodies and probes** | **Company** | **CAT#** | **RRID** |
| --- | --- | --- | --- |
| Anti-centromere antibodies | Antibodies Incorporated | 15-234 |  |
| B23 | Santa Cruz | sc-271737 | AB_10708848 |
| FBXO28 | Bethyl | A302-377A | AB_1907260 |
| FBXO38 | Atlas Antibodies | HPA041444 | AB_2677484 |
| HA | Cell Signaling | 3724 | AB_1549585 |
| Histone H3 | Abcam | ab1791 | AB_302613 |
| PITX2 | Santa Cruz | sc-390457 |  |
| PLZF | Atlas Antibodies | HPA001499 | AB_1079640 |
| SALL4A | Santa Cruz | sc-101147 | AB_1129262 |
| SCP3 | Santa Cruz | sc-74569 | AB_2197353 |
| SKP1 | Cell Signaling | 12248 | AB_2754993 |
| STRA8 | Abcam | ab49405 | AB_945677 |
| Strep II Tag | Novus Biologicals | NBP2-43735 |  |
| Vimentin | Cell Signaling | 5741 | AB_10695459 |
| WT1 | Novus Biologicals | NB110-60011 | AB_905863 |
| ZXDB | Atlas Antibodies | HPA043789 | AB_2678673 |
| α-Tubulin | Proteintech | 66031-1-Ig | AB_11042766 |
| β-Actin | Santa Cruz | sc-69879 | AB_1119529 |
| FSHr (PE Conjugated) | Bioss USA | bs-20658R-PE |  |
| Lectin PNA (AF594 Conjugated) | Thermo Fisher Scientific | L32459 |  |

| **Secondary antibodies** | **Company** | **CAT#** | **RRID** |
| --- | --- | --- | --- |
| Anti-Human IgG (Alexa Fluor 488) | Thermo Fisher Scientific | A11013 | AB_2534080 |
| Anti-Mouse IgG (Alexa Fluor 647) | Abcam | ab150115 | AB_2687948 |
| Anti-Mouse IgG (Alexa Fluor® 555) | Abcam | ab150110 | AB_2783637 |
| Anti-Mouse IgG (DyLight 488) | Thermo Fisher Scientific | 35503 | AB_1965946 |
| Anti-Rabbit IgG (Alexa Fluor® 488) | Abcam | ab150061 | AB_2571722 |
| Anti-Rabbit IgG (Alexa Fluor® 555) | Abcam | ab150070 | AB_2783636 |
| Anti-Rabbit IgG (Alexa Fluor® 647) | Abcam | ab150067 | AB_2894821 |

| **Beads for affinity and immunopurification** | **Company** | **CAT#** |
| --- | --- | --- |
| anti-HA (magnetic) | Pierce | 88837 |
| Strep-Tactin® Superflow resin | IBA | 2-1206-025 |

## Vectors

| **Lentiviral packaging vectors** | **Company** | **Cat#** |
| --- | --- | --- |
| pLenti CMV/TO SV40 Small and Large T antigen | Addgene | 22298 |
| pCMV-dR8.2 | Addgene | 8455 |
| pCMV-VSV-G | Addgene | 8454 |
|  |  |  |
| **cDNA** | **Vector** | **Tag** |
| SALL4 | pcDNA3 | N-terminal HA |
| ZXDA | pcDNA3 | N-terminal HA-tag |
| ZXDA | pcDNA3 | N-terminal SF-tag |
|  |  |  |
| **Sleeping Beauty System** | **Company** | **Cat#** |
| pSBtet-Pur | Addgene | 60507 |
| pSB100X | Addgene | 34879 |

# References

1. Hermann BP, Cheng K, Singh A, Roa-De La Cruz L, Mutoji KN, Chen IC, et al. The Mammalian Spermatogenesis Single-Cell Transcriptome, from Spermatogonial Stem Cells to Spermatids. Cell Rep. 2018;25(6):1650-67 e8.

2. Cordenonsi M, Zanconato F, Azzolin L, Forcato M, Rosato A, Frasson C, et al. The Hippo transducer TAZ confers cancer stem cell-related traits on breast cancer cells. Cell. 2011;147(4):759-72.
